# Regulatory imbalance between LRRK2 kinase, PPM1H phosphatase, and ARF6 GTPase disrupts the axonal transport of autophagosomes

**DOI:** 10.1016/j.celrep.2023.112448

**Published:** 2023-05-01

**Authors:** Dan Dou, Erin M. Smith, Chantell S. Evans, C. Alexander Boecker, Erika L.F. Holzbaur

**Affiliations:** 1 Department of Physiology, Perelman School of Medicine, University of Pennsylvania, Philadelphia, PA 19104, USA; 2 Aligning Science Across Parkinson’s (ASAP) Collaborative Research Network, Chevy Chase, MD 20815, USA; 3 Neuroscience Graduate Group, University of Pennsylvania Perelman School of Medicine, Philadelphia, PA 19104, USA; 4 Duke University Medical Center, Duke University, Durham, NC 27710, USA; 5 Department of Neurology, University Medical Center Goettingen, 37077 Goettingen, Germany; 6 These authors contributed equally; 7 Lead contact

## Abstract

Gain-of-function mutations in the *LRRK2* gene cause Parkinson’s disease (PD), increasing phosphorylation of RAB GTPases through hyperactive kinase activity. We find that LRRK2-hyperphosphorylated RABs disrupt the axonal transport of autophagosomes by perturbing the coordinated regulation of cytoplasmic dynein and kinesin. In iPSC-derived human neurons, knockin of the strongly hyperactive *LRRK2*-p.R1441H mutation causes striking impairments in autophagosome transport, inducing frequent directional reversals and pauses. Knockout of the opposing protein phosphatase 1H (PPM1H) phenocopies the effect of hyperactive LRRK2. Overexpression of ADP-ribosylation factor 6 (ARF6), a GTPase that acts as a switch for selective activation of dynein or kinesin, attenuates transport defects in both p.R1441H knockin and PPM1H knockout neurons. Together, these findings support a model where a regulatory imbalance between LRRK2-hyperphosphorylated RABs and ARF6 induces an unproductive “tug-of-war” between dynein and kinesin, disrupting processive autophagosome transport. This disruption may contribute to PD pathogenesis by impairing the essential homeostatic functions of axonal autophagy.

## In brief

Dou et al. find that the opposing activities of LRRK2 kinase and PPM1H phosphatase dictate a regulatory balance for the motors that drive axonal autophagosome transport. Hyperactive LRRK2, which is associated with Parkinson’s disease (PD), is predicted to contribute to PD pathogenesis by disrupting the essential homeostatic pathway of autophagy.

## Graphical abstract

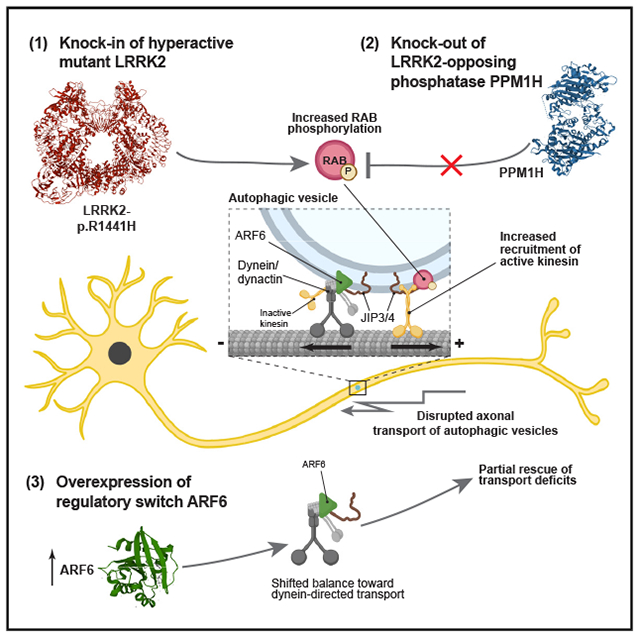

## INTRODUCTION

The leucine-rich repeat kinase 2 (*LRRK2*) gene is a prominent pleomorphic risk locus for Parkinson’s disease (PD), a devastating neurodegenerative disease with debilitating motor and non-motor symptoms. Autosomal dominant missense mutations in *LRRK2* are the most common genetic cause of PD, accounting for ~5% of familial forms.^1^ In addition, genome-wide association studies have linked noncoding variants in *LRRK2* to risk of sporadic PD.

LRRK2 is a large, multidomain enzyme that includes both a ROC-COR tandem GTPase domain and a catalytic kinase domain. Seven gain-of-function pathogenic mutations either directly (mutations located in the kinase domain) or indirectly (mutations located in the ROC or COR domains) increase LRRK2 kinase activity. The most common pathogenic mutation is *LRRK2*-p.G2019S, located in the kinase domain. Three ROC domain mutations at the p.R1441 hotspot (p.R1441C/G/H) decrease LRRK2 GTPase activity, leading to increased kinase activity^2,3^ that exceeds levels induced by the p.G2019S mutation.^2,4^ p.R1441 hotspot mutations may also be more highly penetrant than p.G2019S.^5,6^

LRRK2 phosphorylates a subset of RAB GTPases (RABs), which coordinate intracellular vesicle trafficking by selectively recruiting effector proteins. Phosphorylated RABs have altered interactions with downstream binding partners. These binding partners include the motor adaptor proteins JNK-interacting protein 3 and 4 (JIP3/4), which regulate the activation of the molecular motors cytoplasmic dynein and kinesin on organelle cargos including autophagic vesicles (AVs).^7–9^ Thus, increased LRRK2-mediated phosphorylation of RABs is predicted to alter RAB-dependent intracellular vesicle transport. In recent work, we found that *LRRK2*-p.G2019S decreases the processivity of retrograde axonal AV transport by recruiting JIP4 and inducing inappropriate kinesin activity.^10,11^ While LRRK2 activity leads to increased RAB phosphorylation, protein phosphatase 1H (PPM1H) has recently been found to counteract this effect by specifically dephosphorylating LRRK2-phosphorylated RAB GTPases.^12^

The transport of AVs in neurons is of particular importance for the maintenance of axonal homeostasis. AVs are formed preferentially in the distal axon and at presynaptic sites before undergoing processive retrograde motility toward the soma, driven primarily by the minus-end-directed microtubule-associated motor protein cytoplasmic dynein.^13–17^ The opposing plus-end-directed kinesin motor remains bound to AVs during dynein-driven transport but is auto-inhibited under normal conditions.^10,13,18^ During retrograde transport, the AV fuses with one to two lysosomes, lowering the intra-lumenal pH and activating degradative enzymes.^19^ Importantly, AV transport is tightly linked to AV maturation and function in cargo degradation.^20–22^

Here, we demonstrate that the opposing activities of LRRK2 kinase and PPM1H phosphatase dictate an important regulatory balance for the motor proteins that drive autophagosome transport. We found that neurons expressing the highly hyperactive *LRRK2*-p.R1441H mutation exhibited a more severe transport deficit than was previously observed in p.G2019S knockin (KI) neurons,^10^ suggesting that increased disruptions in autophagosome transport scale with higher levels of LRRK2 kinase activity. In complementary experiments, we found that knockout (KO) of PPM1H also impaired AV transport, while PPM1H overexpression rescued AV transport in neurons expressing hyperactive LRRK2, confirming the model that balanced kinase and phosphatase activities are required for normal AV transport. Finally, we found that overexpression of ADP-ribosylation factor 6 (ARF6), which acts as a switch to regulate the association of the activating adaptor JIP3/4 with either dynein/dynactin or kinesin,^23^ attenuated defective AV transport in both p.R1441H KI and PPM1H KO neurons. Thus, our data indicate that the balanced interplay among ARF6 activity, JIP3/4 association, and RAB phosphorylation state permits unopposed retrograde autophagosome transport in wild-type (WT) neurons, while hyperactivation of LRRK2 by PD-associated mutations leads to a regulatory imbalance, resulting in an inappropriate “tug-of-war” between dynein and kinesin. Our data robustly link hyperphosphorylation of RABs to defects in neuronal autophagy, an essential homeostatic pathway that has long been implicated in PD pathogenesis.

## RESULTS

### Endogenous LRRK2-p.R1441H disrupts the axonal transport of autophagosomes in iPSC-derived neurons

A point mutation in the kinase domain of LRRK2 (*LRRK2*-p.G2019S) was previously determined to disrupt the axonal transport of AVs in NGN2-induced human neurons (iNeurons) that express endogenous LRRK2.^10,24,25^ Mutations in the p.R1441 hotspot located in the ROC domain of LRRK2 also cause PD, potentially with higher penetrance.^5,26,27^ Consistent with previous reports, we found that *LRRK2*-p.R1441C causes a higher magnitude of kinase hyperactivity than *LRRK2*-p.G2019S in KI mouse embryonic fibroblasts (MEFs, Figure S1). Using a pan-specific antibody for phosphorylated RAB3A, 8A, 10, 35, and 43, we measured a ~2.5-fold increase in RAB phosphorylation over WT for p.R1441C compared with ~1.5-fold for p.G2019S, representing ~66% greater hyperactivity of the p.R1441C mutation (Figures S1A and S1B). As expected, the hyperactive kinase activity induced by either the p.R1441C or p.G2019S mutation was effectively inhibited by the LRRK2 kinase inhibitor MLi-2. When measuring specifically phosphorylation of RAB10, we similarly observed greater hyperactivity of *LRRK2*-p.R1441C compared with the p.G2019S mutation (Figure S1C).

Given the higher level of hyperactivity induced by an R1441-hotspot mutant, we hypothesized that these mutations would more potently disrupt the axonal transport of AVs. To test this hypothesis, we used human induced pluripotent stem cells (iPSCs) from the KOLF2.1J parental line that were gene-edited to a heterozygous KI of the *LRRK2*-p.R1441H mutation by the iPSC Neurodegenerative Disease Initiative (iNDI) at the NIH.^28^ We differentiated these iPSCs into excitatory glutamatergic neurons using tetracycline-inducible expression of NGN2.^29^ Consistent with our findings in the MEF system, we observed increased RAB10 phosphorylation in *LRRK2*-p.R1441H KI iNeurons compared to isogenic control neurons using both western blot and immunostaining approaches (Figures S2A and S2C), which was reversed by treatment with the LRRK2 inhibitor MLi-2.^30^ In contrast, we were unable to reliably detect increased pRAB10 levels in p.G2019S KI iNeurons versus paired isogenic control neurons, possibly due to low signal-to-noise in assessing pRAB levels in neurons, as has been previously suggested.^12,31–33^

**Figure 1.**
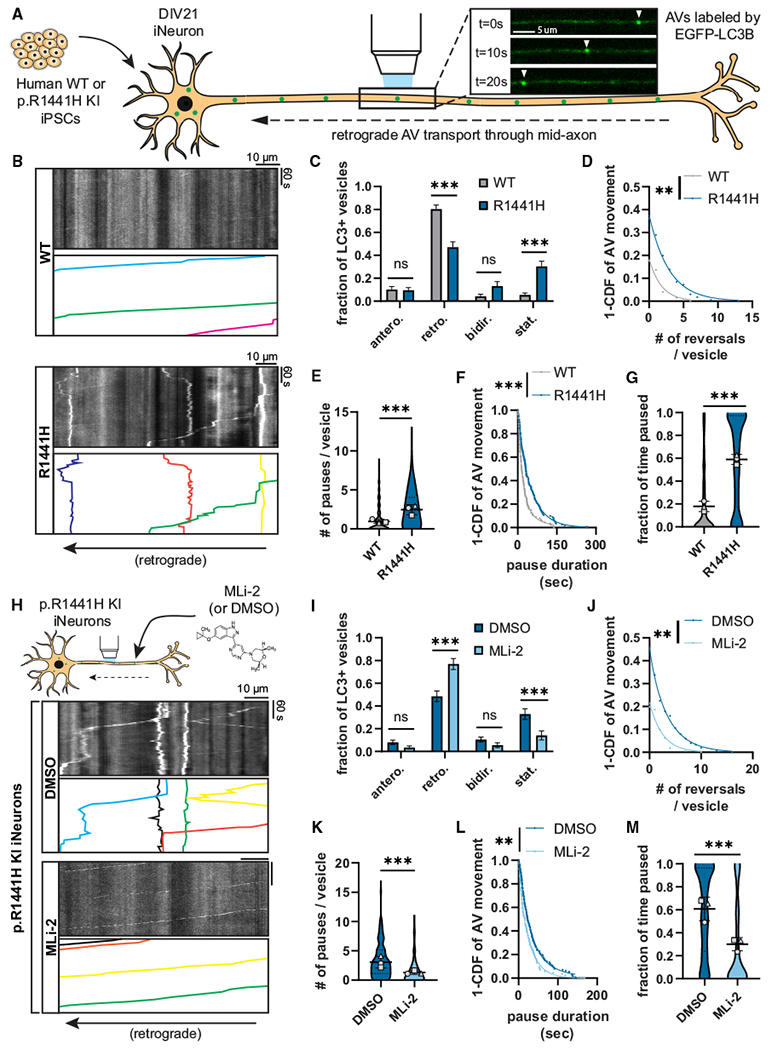
*LRRK2*-p.R1441H knockin causes kinase-dependent disruption of axonal AV transport (A) Example time lapse images of EGFP-LC3+ vesicles in the mid-axon of a WT iPSC-derived neuron (iNeuron). (B) Kymographs of axonal EGFP-LC3+ vesicles in WT and p.R1441H KI iNeurons. Example AV traces are highlighted. (C) Directionality of AVs in WT and p.R1441H KI iNeurons. Antero., anterograde; retro., retrograde; bidir., bidirectional; stat., stationary (mean ± SEM; n = 31 neurons from three independent experiments; ns, not significant, p > 0.2026; ****p < 0.001; two-way ANOVA with Sidak’s multiple comparisons test). (D–F) Directional reversals (D), pause number (E), and pause duration (F) of motile AVs in WT and p.R1441H KI iNeurons (mean ± SD for E; n = 168–200 motile AVs from 31 neurons from three independent experiments; **p = 0.00581; ***p < 0.001; mixed effects model analysis, see STAR Methods for specific models used). (G) Fraction of time paused of all AVs in WT and p.R1441H KI iNeurons (mean ± SD; n = 209–246 AVs from 31 neurons from three independent experiments; ***p < 0.001; mixed effects model analysis). (H) Kymographs of axonal EGFP-LC3+ vesicles in p.R1441H KI iNeurons treated overnight with DMSO or 100 nM MLi-2. (I) Directionality of AVs in p.R1441H iNeurons treated with DMSO or MLi-2 (mean ± SEM; n = 30 neurons from three independent experiments; ns, not significant, p > 0.7779; ***p < 0.001; two-way ANOVA with Sidak’s multiple comparison test). (J–L) Directional reversals (J), pause number (K), and pause duration (L) of motile AVs in p.R1441H KI iNeurons treated with DMSO or MLi-2 (mean ± SD for J; n = 153–163 motile AVs from 30 neurons from three independent experiments; **p < 0.00723; ***p < 0.001; mixed effects model analysis). (M) Fraction of time paused of AVs in p.R1441H KI iNeurons treated with DMSO or MLi-2 (mean ± SD; n = 190–221 AVs from 30 neurons from three independent experiments; ***p < 0.001; mixed effects model analysis). For (E), (G), (K), and (M), scatterplot points indicate the means of three independent experiments. For (D), (F), (J), and (L), curve fits were generated using nonlinear regression (two-phase decay). See also Figures S1 and S2 and Video S1.

To assess axonal AV transport in these NGN2-induced p.R1441H KI iNeurons, we live-imaged EGFP-LC3-labeled AVs in the mid-axon at DIV21 (Figure 1A; Video S1). We found that the expression of *LRRK2*-p.R1441H caused striking changes in the dynamics of axonal AV transport (Figure 1B). When scoring the motility and directionality of AV movement (classifications defined in Figure S2D), we found a marked increase in the stationary fraction of AVs at the expense of the retrograde fraction (Figure 1C). This change in the net directionality of AVs along the axon is in marked contrast to our previous studies on p.G2019S KI iNeurons, where we noted significantly increased pausing of AVs moving along the axon but no change in the overall fraction of retrograde organelles.^10^

To further explore which transport parameters might be disrupted by *LRRK2*-p.R1441H, we quantified non-processive motility and pausing behavior of motile AVs in p.R1441H KI vs. isogenic WT iNeurons. p.R1441H KI neurons exhibited reduced AV processivity, with higher number of directional reversals (Figure 1D) and significantly increased Δ run length, a metric of non-processive motility calculated as the difference between the total and net run length of each vesicle (Figure S2F; Δ run length defined in S2E). We also observed an ~3-fold increase in the number of pauses per vesicle in p.R1441H KI iNeurons (Figure 1E). While this effect was of similar magnitude to that previously observed in p.G2019S KI iNeurons, the average pause duration was significantly increased in p.R1441H KI iNeurons (Figure 1F) but not in p.G2019S KI iNeurons.^10^

Thus, expression of the p.R1441H mutation severely disrupted axonal AV transport, with an increased fraction of stationary AVs, an increased number of pauses, and an increase in the pause duration of motile AVs. Combined, these factors manifested as a striking increase in the overall fraction of time that p.R1441H KI AVs spent paused. AVs in p.R1441H KI iNeurons paused for nearly 60% of the time, representing a 3.2-fold increase over WT (Figure 1G). The same quantification performed for AV transport in p.G2019S KI iNeurons (Boecker et al., 2021^10^; data not shown) showed AVs paused for ~40% of the time (1.97-fold over WT). When normalized to corresponding isogenic WT iNeurons, the p.R1441H effect exceeded that of p.G2019S by ~61%, appearing to closely correlate with the ~66% difference in kinase hyperactivity we observed between p.G2019S and a p.R1441 hotspot mutation.

To confirm whether the observed AV transport deficits are LRRK2 kinase activity dependent, we next tested whether the selective LRRK2 inhibitor MLi-2^30^ rescues AV transport in p.R1441H KI iNeurons (Figure 1H). Overnight treatment of iNeurons with 100 nM MLi-2 significantly increased the retrograde fraction of AVs at the expense of the stationary fraction (Figure 1I). Non-processive motility in p.R1441H KI neurons was also rescued, as measured by a reduction of the number of directional reversals and Δ run length (Figures 1J and S2G). Pharmacological LRRK2 inhibition with MLi-2 also reduced pause number and pause duration of motile AVs (Figures 1K and 1L), rescuing the overall fraction of time spent paused for axonal AVs in p.R1441H KI neurons (Figure 1M). Together, these results are consistent with our previously proposed model of a pathogenic “tug-of-war” between anterograde and retrograde motor proteins induced by hyperactive LRRK2.^10^ Importantly, our data also suggest that disruption of AV transport scales in a kinase activity-dependent manner, with greater severity of AV transport defects observed upon expression of p.R1441H compared with p.G2019S.

### Overexpression of the LRRK2-counteracting phosphatase PPM1H rescues axonal AV transport in p.R1441H KI and p.G2019S KI neurons

Recent work has identified PPM1H as the phosphatase counteracting LRRK2 activity through specific dephosphorylation of RAB GTPases.^12^ In HEK293 cells transiently expressing LRRK2-p.R1441G, overexpression of GFP-PPM1H^WT^ resulted in reduced levels of phosphothreonine RABs compared to overexpression of the catalytically inactive mutant GFP-PPM1H^H153D^ (Figure S3A).^12^ Transient expression of GFP-PPM1H^WT^ in p.G2019S KI MEFs had a similar effect, despite a low transfection efficiency (Figure S3B).

**Figure 2.**
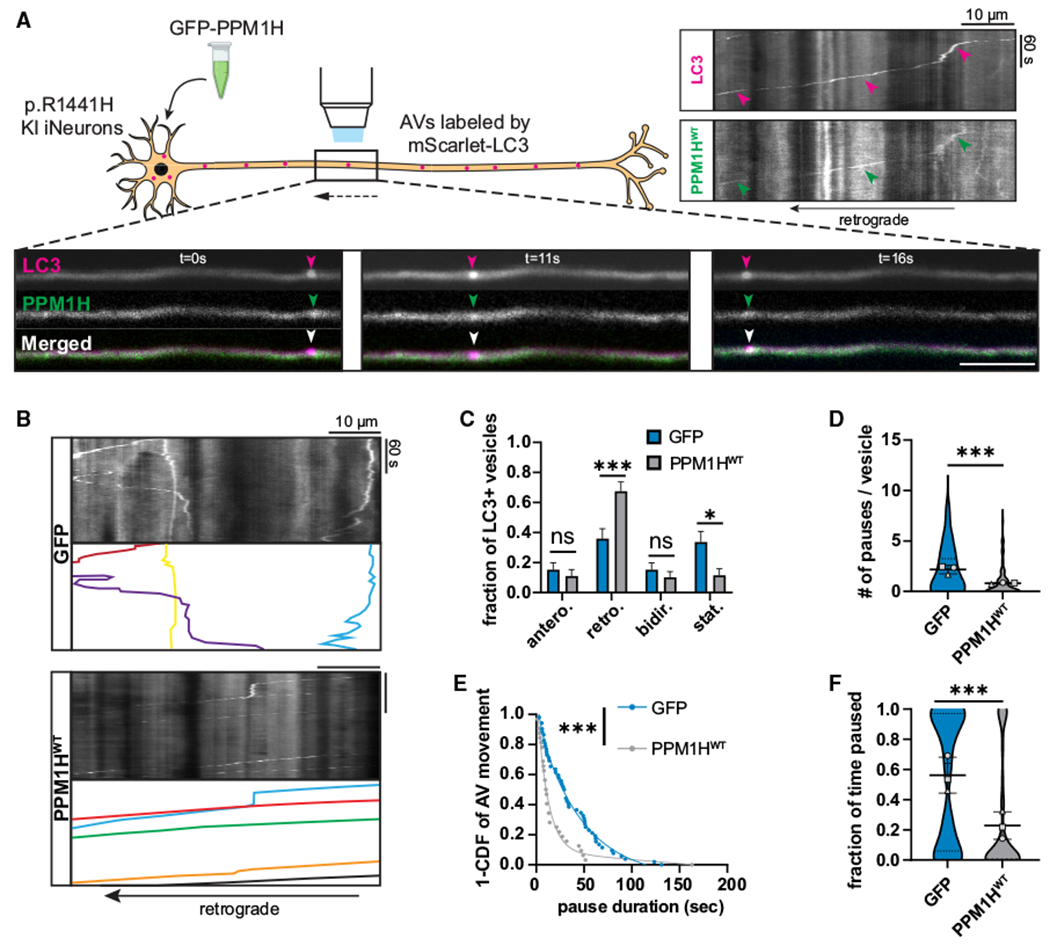
Overexpression of PPM1H rescues AV transport in neurons expressing hyperactive LRRK2 (A) Top right, kymographs of axonal mScarlet-LC3+ and GFP-PPM1H^WT^+ vesicles in a p.R1441H KI iNeuron. Magenta arrowheads point to track of mScarlet-LC3+ vesicle, and green arrowheads highlight GFP-PPM1H^WT^+ track that colocalizes with mScarlet-LC3+ track. Below, time lapse images of axonal mScarlet-LC3+ and GFP-PPM1H^WT^+ vesicles in a p.R1441H KI iNeuron. Scale bar, 10 μm. (B) Kymographs of axonal mScarlet-LC3+ vesicles in p.R1441H KI iNeurons co-expressing GFP or GFP-PPM1H^WT^. Example AV traces are highlighted. (C) Directionality of AVs in p.R1441H KI iNeurons co-expressing GFP or GFP-PPM1H^WT^. Antero., anterograde; retro., retrograde; bidir., bidirectional; stat., stationary (mean ± SEM; n = 19–20 neurons from three independent experiments; ns, not significant, p > 0.9382; *p = 0.0164; ***p = 0.0002; two-way ANOVA with Sidak’s multiple comparisons test). (D and E) Pause number (D) and pause duration (E) of motile AVs in p.R1441H KI iNeurons co-expressing GFP or GFP-PPM1H^WT^ (mean ± SD for D; n = 82–86 motile AVs from 19 to 20 axons from three independent experiments; ***p < 0.001; mixed effects model analysis). (F) Fraction of time paused of all AVs in p.R1441H KI iNeurons co-expressing GFP or GFP-PPM1H^WT^ (mean ± SD; n = 104–126 AVs from 19 to 20 axons from three independent experiments; ***p < 0.001; mixed effects model analysis). For (D) and (F), scatterplots indicate the means of three independent experiments. For (E), curve fit was generated using nonlinear regression (two-phase decay). See also Figure S3 and Videos S2 and S3.

We asked whether enhanced RAB dephosphorylation by PPM1H overexpression could reverse AV transport disruption in neurons expressing hyperactive LRRK2. First, we investigated whether PPM1H^WT^ overexpression could reverse the changes in AV transport observed in human *LRRK2*-p.R1441H KI iNeurons. Visualizing AV transport in the mid-axon with mScarlet-LC3, we observed cotransport of GFP-PPM1H^WT^ with LC3-positive AVs along the axon (Figure 2A). Transient overexpression of GFP-PPM1H^WT^ significantly improved retrograde AV transport in p.R1441H KI iNeurons, while overexpression of GFP alone had no effect (Figure 2B). We observed a significant increase in the retrograde fraction of AVs at the expense of the stationary fraction (Figure 2C), as well as reduced pause number (Figure 2D), pause duration (Figure 2E), and fraction of time spent paused for axonal AVs (Figure 2F). In contrast, we did not observe a significant change in directional reversals or Δ run lengths in PPM1H-overexpressing p.R1441H KI iNeurons compared to WT control neurons, suggesting that rescue of the transport phenotype was not complete.

Next, we performed parallel experiments in primary cortical neurons from *Lrrk2*-p.G2019S KI mice (Figure S3C, Video S2), comparing the expression of GFP, GFP-PPM1H, or the catalytically inactive variant GFP-PPM1H^H153D^. Notably, PPM1H^WT^ overexpression improved retrograde AV processivity in p.G2019S KI neurons compared with either GFP mock or PPM1H^H153D^ transfection (Figure S3D, Video S3). Overexpression of GFP-PPM1H^WT^ significantly decreased the number of pauses, pause duration, and overall fraction of time paused of AVs in p.G2019S KI neurons (Figures S3E–S3G). GFP-PPM1H^WT^ overexpression also reduced non-processive AV motility as quantified by the number of directional reversals and Δ run length (Figures S3H and S3I). However, we did not observe an effect on AV directionality from PPM1H overexpression in p.G2019S KI mouse cortical neurons (Figure S3J). Together, these data from both human iNeurons and mouse cortical neurons implicate a causal role for RAB hyperphosphorylation in the disruption of AV transport by hyperactive LRRK2, which can be partially reversed by exogenous expression of active PPM1H phosphatase.

### PPM1H KO phenocopies the effect of hyperactive LRRK2 on AV transport

**Figure 3.**
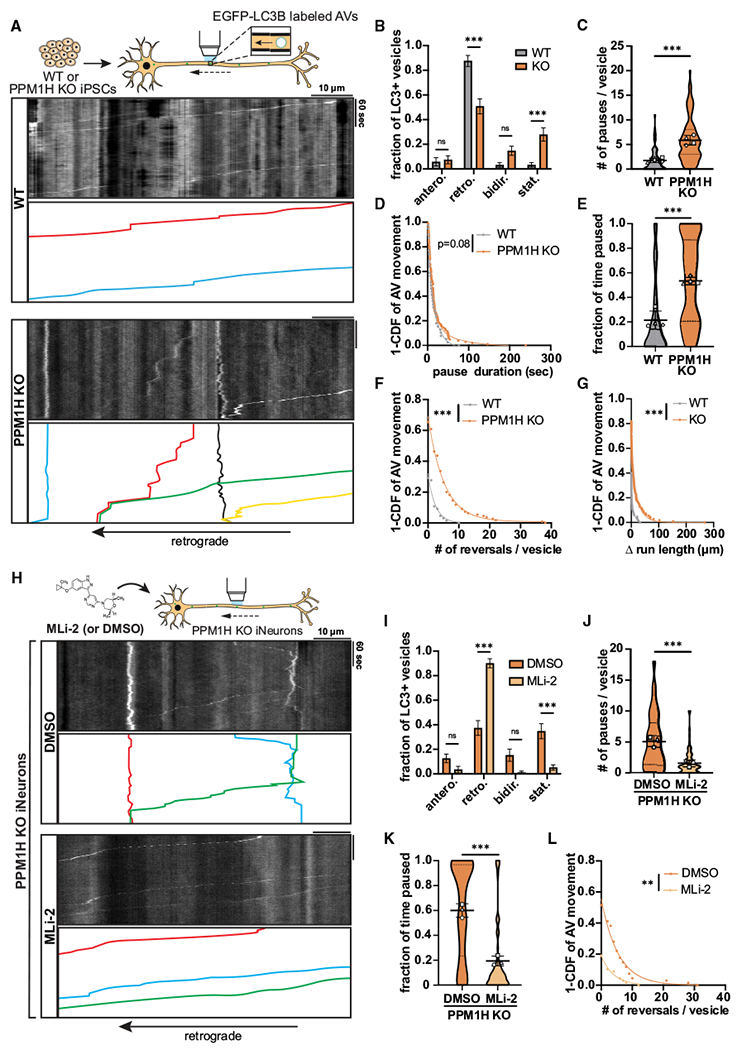
Knockout of PPM1H causes LRRK2 kinase-dependent disruption of axonal AV transport (A) Kymographs of axonal EGFP-LC3+ vesicles in WT and PPM1H KO iNeurons. Example AV traces are highlighted. (B) Directionality of AVs in WT and PPM1H KO iNeurons. Antero., anterograde; retro., retrograde; bidir., bidirectional; stat., stationary (mean ± SEM; n = 32–39 neurons from four independent experiments; ns, not significant, p > 0.1996; ***p < 0.001; two-way ANOVA with Sidak’s multiple comparisons test). (C–G) Pause number (C) and pause duration (D) of motile AVs, fraction of time paused (E) of all AVs, and directional reversals (F) and Δ run length (G) of motile AVs in WT and PPM1H KO iNeurons (mean ± SD for C and E; n = 65–110 motile AVs for C, D, F, and G and 69–141 total AVs for E from 32 to 39 neurons from four independent experiments; ***p < 0.001; mixed effects model analysis). (H) Kymographs of axonal EGFP-LC3+ vesicles in PPM1H KO iNeurons treated over 72 h with DMSO or 200 nM MLi-2. Example AV traces are highlighted. (I) Directionality of AVs in PPM1H KO iNeurons treated with DMSO or MLi-2 (mean ± SEM; n = 28–30 neurons from three independent experiments; ns, not significant, p > 0.0772; ***p < 0.001; two-way ANOVA with Sidak’s multiple comparison test). (J–L) Pause number (J) of motile AVs, fraction of time paused (K) of all AVs, and directional reversals (L) of motile AVs in PPM1H KO iNeurons treated with DMSO or MLi-2 (mean ± SD for J and K; n = 68 motile AVs for J and Land 73–104 total AVs for K from 28 to 30 neurons from three independent experiments; **p = 0.00118; ***p < 0.001; mixed effects model analysis). For (C), (E), (J), and (K), scatterplot points indicate the means of three independent experiments. For (D), (F), (G), and (L), curve fits were generated using nonlinear regression (two-phase decay). See also Figure S4 and Video S4.

To confirm the detrimental effect of RAB hyperphosphorylation on axonal AV transport, we next investigated whether PPM1H KO phenocopies the impairment of AV transport by expression of hyperactive LRRK2. Using the KOLF2.1J parental line, we generated PPM1H KO iPSCs through CRISPR-Cas9 gene editing and differentiated them into excitatory glutamatergic iNeurons (Figure S4A). Western blot with a pan-specific antibody for phosphorylated RAB3A, 8A, 10, 35, and 43 showed elevated levels of LRRK2-phosphorylated RAB proteins in PPM1H KO iNeurons (Figure S4B), with similar results from western blot and immunostaining with antibodies specific for LRRK2-phosphorylated RAB10 (Figures S2A and S2C). Similar to expression of the p.R1441H mutation in LRRK2, PPM1H KO dramatically affected AV directionality, with an increase of the stationary fraction at the expense of the retrograde fraction (Figures 3A and 3B, Video S4). Furthermore, motile AVs in PPM1H KO iNeurons showed a significant increase in the number of pauses as well as a trend toward increased pause duration that did not achieve statistical significance (Figures 3C and 3D). Together, increased pausing and number of stationary AVs resulted in a marked increase of the overall fraction of time that PPM1H KO AVs spent paused, reaching a similar level as observed in p.R1441H KI iNeurons (Figure 3E). PPM1H KO also phenocopied the effect of hyperactive LRRK2 on non-processive AV motility, increasing the number of directional reversals and Δ run length of motile AVs (Figures 3F and 3G).

To verify whether PPM1H KO and LRRK2 hyperactivity disrupt AV transport through the same pathway, we applied MLi-2 to PPM1H KO iNeurons to inhibit LRRK2 kinase activity (Figure 3H). Compared with DMSO control treatment, MLi-2 rescued AV directionality, with retrograde motility restored to the previously stationary fraction (Figure 3I). MLi-2 furthermore decreased the number of pauses and pause duration of motile AVs, restoring the overall fraction of time paused to levels similar to WT iNeurons (Figures 3J, 3K, and S4C). Non-processive motility of motile AVs, as measured by number of directional reversals and Δ run length, was also rescued by LRRK2 inhibition (Figures 3L and S4D). In sum, these results from orthogonal PPM1H model systems provide further evidence that RAB GTPase hyperphosphorylation induces disruption of processive retrograde AV transport.

### Hyperactive LRRK2 does not disrupt axonal transport of mitochondria

If pathogenic LRRK2 mutations disrupt axonal transport via hyperphosphorylation of RAB proteins, we would predict that the effect would be cargo specific, such that cargos whose transport is not regulated by RABs would not be similarly affected. To this end, we tested whether hyperactive LRRK2 affects the axonal transport of mitochondria, a cargo that undergoes both anterograde and retrograde active transport driven by cytoplasmic dynein and kinesin-1 motors but with no known role for RAB GTPases in regulation of their motility.^34^ Mitochondrial transport in the axon is also of specific interest given the established role of mitochondrial dysfunction in PD pathophysiology.^35^

**Figure 4.**
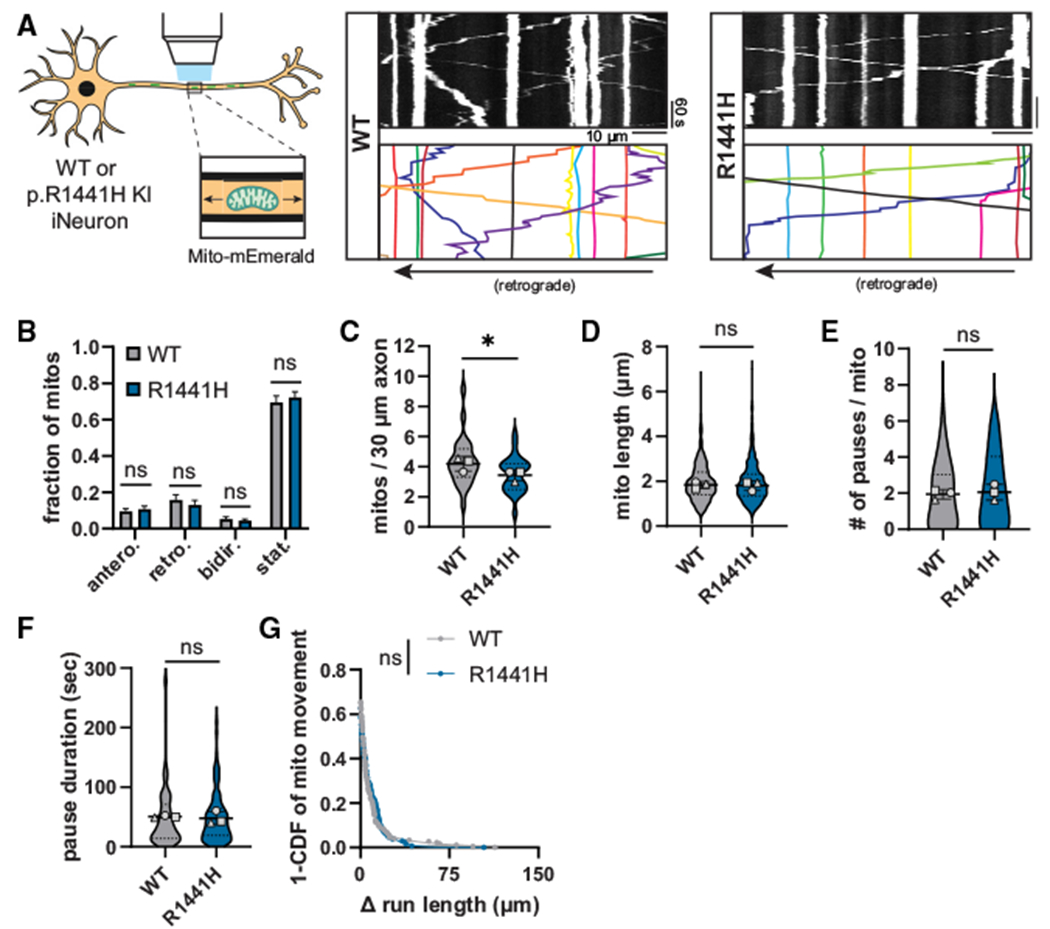
*LRRK2*-p.R1441H does not disrupt axonal mitochondria transport in human iNeurons (A) Kymographs of axonal mitochondria labeled by Mito-mEmerald in WT and p.R1441H KI iNeurons. Example mitochondrial traces are highlighted. (B) Directionality of mitochondria in WT and p.R1441H KI iNeurons. Antero., anterograde; retro., retrograde; bidir., bidirectional; stat., stationary (mean ± SEM; n = 26 neurons from three independent experiments; ns, not significant, p > 0.8700; two-way ANOVA with Sidak’s multiple comparisons test). (C and D) Density per 30-μm axon (C) and average length (D) of total population of mitochondria in WT and p.R1441H KI iNeurons (mean ± SD; n = 483–571 mitochondria from 26 neurons from three independent experiments; ns, not significant, p = 0.2918; *p = 0.0348; mixed effects model analysis). (E–G) Pause number (E), pause duration (F), and Δ run length (G) of mitochondria in WT and p.R1441H KI iNeurons (mean ± SD for E and F; n = 136–181 motile mitochondria from 26 neurons from three independent experiments; ns, not significant, p > 0.492; mixed effects model analysis). For (C)–(F), scatterplot points indicate the means of three independent experiments. For (G), a curve fit was generated using nonlinear regression (two-phase decay). See also Figures S5 and S6 and Video S5.

We visualized mitochondrial transport in the mid-axon of DIV21 p.R1441H KI iNeurons and isogenic WT neurons with Mito-mEmerald (Figure 4A, Video S5). Similar to our previous characterization of DIV21 iNeurons from a different parental iPSC line,^36^ ~75% of mitochondria in the mid-axon were stationary or bidirectional in both WT and p.R1441H KI iNeurons. Expression of the p.R1441H mutation neither altered the fraction of motile mitochondria nor perturbed directionality (Figure 4B). We observed a statistically significant but small decrease in axonal mitochondrial density in p.R1441H KI neurons, while there was no change in average mitochondrial length (Figures 4C and 4D), suggesting that the fission/fusion balance of the mitochondrial network was mostly unchanged.

To investigate whether LRRK2-p.R1441H disrupts transport of the motile (anterograde, retrograde, and bidirectional) axonal population of mitochondria, we excluded stationary mitochondria from further analysis. We observed no differences in pause number, pause duration, or Δ run length between WT and p.R1441H KI neurons (Figures 4E–4G). Furthermore, there were no direction-specific effects on either the anterograde or retrograde population (Figures S5A–S5D).

We performed similar live imaging of mitochondrial transport in DIV21 *LRRK2*-p.G2019S KI iNeurons and isogenic WT neurons (Figure S6A). In p.G2019S KI neurons, we observed a small but statistically significant decrease in the stationary fraction of mitochondria, but no change in the anterograde, retrograde, or bidirectional fractions (Figure S6B). Axonal mitochondrial density in p.G2019S KI neurons was slightly higher than in WT neurons (Figure S6C), contrasting with the small decrease in mitochondrial density in p.R1441H KI neurons (Figure 4C) and thus suggesting that these changes are unlikely to be a biologically significant effect of LRRK2 hyperactivity. There was no change in average mitochondrial length (Figure S6D). Similar to our observations with p.R1441H KI neurons, no changes in pause number, pause duration, or Δ run length of the motile population of axonal mitochondria were observed in p.G2019S KI neurons (Figures S6E–S6G). Likewise, p.G2019S KI neurons did not display direction-specific effects on either the anterograde or retrograde population (Figures S5E–S5H).

In addition to human iNeurons, we also investigated the effect of hyperactive LRRK2 on mitochondrial transport in primary cortical neurons from *Lrrk2*-p.G2019S KI mice. We used Mito-SNAP to visualize mitochondrial transport in the mid-axon of WT and p.G2019S KI mouse cortical neurons treated with DMSO or 100 nM MLi-2 overnight (Figure S6H). We observed no changes in axonal density, average length, or directionality of mitochondria from either p.G2019S KI or MLi-2 application (Figures S6I–S6K).

In summary, we found that neither the ROC domain mutation *LRRK2*-p.R1441H nor the kinase domain mutation *LRRK2*-p.G2019S affects axonal mitochondrial transport under basal conditions. Together with our previous observations that *LRRK2*-p.G2019S KI does not impair transport of LAMP1-positive vesicles,^10^ these results underscore the finding that LRRK2 hyperactivity does not indiscriminately disrupt axonal transport, but rather it has a cargo-selective effect on AVs.

### Overexpression of the small GTPase ARF6 ameliorates pRAB-mediated disruption of AV transport

Processive retrograde AV transport depends on the tight regulation of the opposing activities of two microtubule-associated motor proteins stably bound to autophagosomes. Both the anterograde motor kinesin-1 and the retrograde motor cytoplasmic dynein (in complex with dynactin) are bound to AVs, but adaptor proteins inhibit kinesin and promote dynein activity in WT neurons.^13,17,22,37^ In our previous work, we found that hyperactive LRRK2 recruits the motor adaptor JIP4, which specifically binds to LRRK2-phosphorylated RAB proteins, to the AV membrane.^7–10^ This results in abnormal recruitment and activation of kinesin that disrupts processive retrograde AV transport.^10^

**Figure 5.**
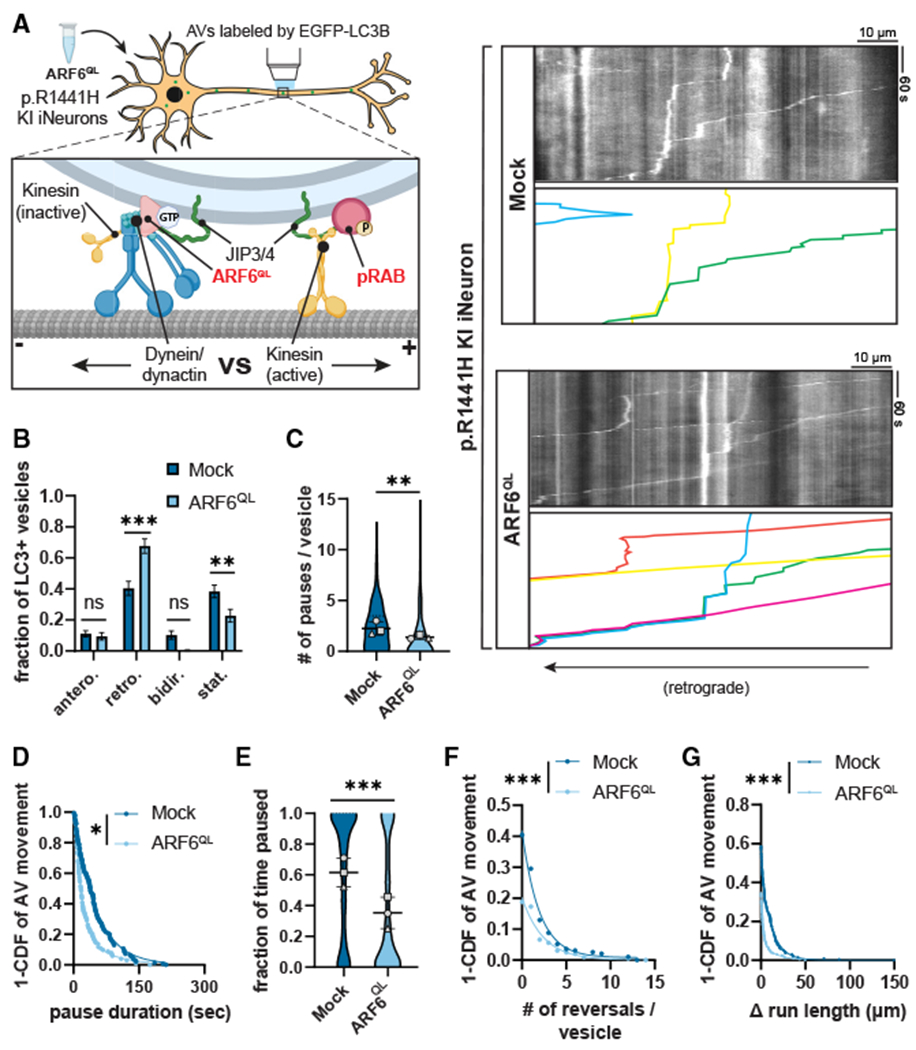
Overexpression of ARF6^Q67L^ ameliorates AV transport deficits in *LRRK2*-p.R1441H KI iNeurons (A) Kymographs of axonal EGFP-LC3+ vesicles in p.R1441H KI iNeurons transiently expressing CFP (“Mock”) or ARF6^Q67L^-CFP. Example AV traces are highlighted. Cartoon depicts competition between ARF6^Q67L^ and phospho-RAB for binding with JIP4, with opposing directionality of binding partner motors. (B) Directionality of AVs in p.R1441H KI iNeurons transiently expressing CFP or ARF6^Q67L^-CFP. Antero., anterograde; retro., retrograde; bidir., bidirectional; stat., stationary (mean ± SEM; n = 27–28 neurons from three independent experiments; ns, not significant, p > 0.1492; **p = 0.0053; ***p < 0.0001; two-way AN OVA with Sidak’s multiple comparisons test). (C and D) Pause number (C) and pause duration (D) of motile AVs in p.R1441H KI iNeurons transiently expressing CFP or ARF6^Q67L^-CFP (mean ± SD for C; n = 195–199 motile AVs from 27 to 28 neurons from three independent experiments; *p = 0.0123; **p =0.00261; mixed effects model analysis). (E) Fraction of time paused of all AVs in p.R1441H KI iNeurons transiently expressing CFP or ARF6^Q67L^-CFP (mean ± SD; n = 257–321 AVs from 27 to 28 neurons from three independent experiments; ***p < 0.001; mixed effects model analysis). (F and G) Directional reversals (F) and Δ run length (G) of motile AVs in p.R1441H KI iNeurons transiently expressing CFP or ARF6^Q67L^-CFP (n = 195–199 motile AVs from 27 to 28 neurons from three independent experiments; ***p < 0.001; mixed effects model analysis). For (C) and (E), scatterplot points indicate the means of three independent experiments. For (D), (F), and (G), curve fits were generated using nonlinear regression (two-phase decay). See also Video S6.

The active, GTP-bound form of the small GTPase ARF6 binds to the leucine zipper II domain of JIP4 or its closely related paralog JIP3, promoting interaction of JIP3/4 with dynactin while inhibiting the JIP-kinesin interaction.^23^ Our group recently found that overexpression of GTP-locked ARF6^Q67L^ did not affect axonal AV transport in WT rat hippocampal neurons, but expression of GDP-locked ARF6^T27N^ decreased retrograde motility of axonal AVs, supporting a key role for ARF6 in the regulation of AV transport.^38^ Notably, while JIP3/4 levels were increased on AVs isolated from mice expressing hyperactive LRRK2, ARF6 levels were not.^10^ We hypothesized that ARF6 levels become limiting in the presence of hyperphosphorylated RABs and that overexpression of GTP-ARF6 may restore retrograde AV motility by counterbalancing the abnormally increased levels of JIP3/4 on the AV membrane (depicted in cartoon in Figure 5A).

To test this model, we transiently expressed the GTP-locked mutant ARF6^Q67L^-CFP in DIV21 *LRRK2*-p.R1441H KI iNeurons (Figure 5A, Video S6) and visualized AV transport in the mid-axon with EGFP-LC3B. In the CFP mock condition, we again observed dramatic disruption of retrograde AV transport associated with p.R1441H KI. However, overexpression of ARF6^QL^ significantly ameliorated this disruption, increasing the retrograde fraction of AVs at the expense of the stationary fraction (Figure 5B). ARF6^QL^ expression also reduced pausing behavior of motile AVs, reflected by decreases in number of pauses and pause duration (Figures 5C and 5D). Reduced pausing and stationary fraction resulted in an overall decrease in the fraction of time p.R1441H KI AVs spent paused (Figure 5E). Non-processive motility was also decreased by ARF6^QL^ expression, as quantified by directional reversals and Δ run length (Figures 5F and 5G). For each metric of AV transport, ARF6^QL^-expressing iNeurons approached but did not reach WT levels (Figure 1), consistent with a partial rescue of the phenotype in p.R1441H KI neurons.

**Figure 6.**
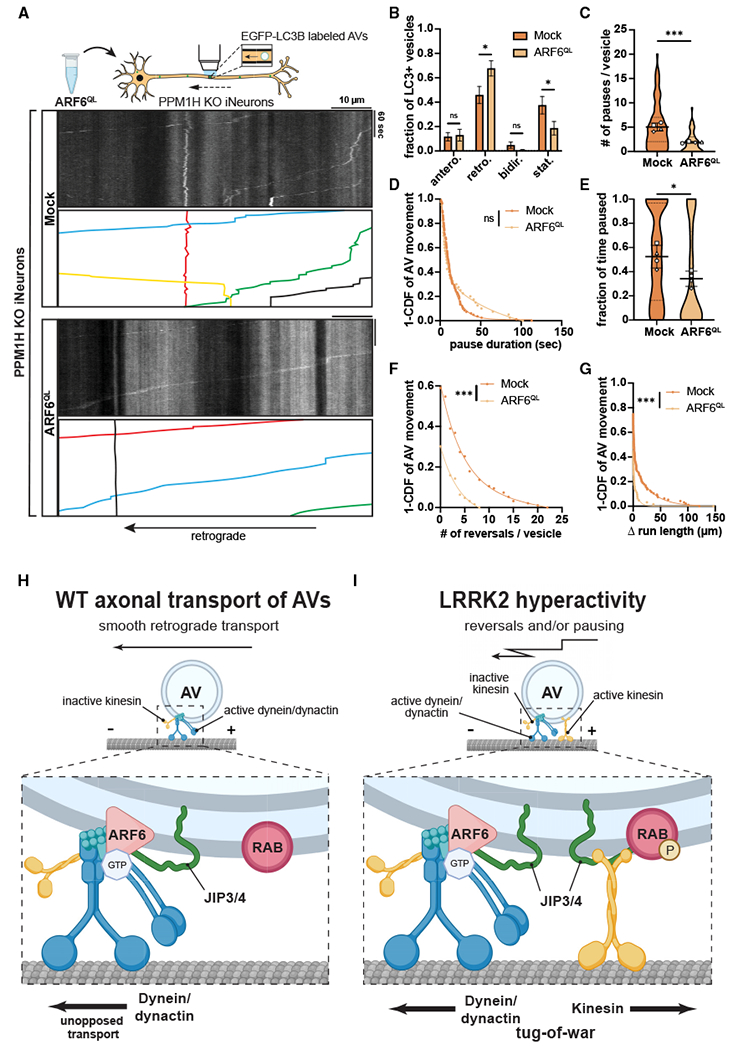
Overexpression of ARF6^Q67L^ ameliorates AV transport deficits in PPM1H KO iNeurons (A) Kymographs of axonal EGFP-LC3+ vesicles in PPM1H KO iNeurons transiently expressing CFP (“Mock”) or ARF6^Q67L^-CFP. Example AV traces are highlighted. (B) Directionality of AVs in PPM1H KO iNeurons transiently expressing CFP or ARF6^Q67L^-CFP. Antero., anterograde; retro., retrograde; bidir., bidirectional; stat., stationary (mean ± SEM; n = 32–34 neurons from four independent experiments; ns, not significant, p > 0.9652; *p < 0.0410; two-way ANOVA with Sidak’s multiple comparisons test). (C–G) Pause number (C) and pause duration (D) of motile AVs, fraction of time paused (E) of all AVs, and directional reversals (F) and Δ run length (G) of motile AVs in PPM1H KO iNeurons transiently expressing CFP or ARF6^Q67L^-CFP (mean ± SD for C and E; n = 63–95 motile AVs for C, D, F, and G and 77–134 total AVs for E from 32 to 34 neurons from four independent experiments; ns, not significant, p = 0.638; *p = 0.0396; ***p < 0.001; mixed effects model analysis). For (C) and (E), scatterplot points indicate the means of four independent experiments. For panels (D), (F), and (G), curve fits were generated using nonlinear regression (two-phase decay). (H and I) Model: imbalance between ARF6 and pRABs disrupts axonal AV transport in conditions of LRRK2 hyperactivity. (H) Under physiologic conditions, ARF6 binds to JIP4, promoting JIP4 binding to dynactin while discouraging the JIP4-kinesin interaction. With active dynein/dynactin and inactive kinesin, AVs undergo smooth retrograde transport in WT neurons. (I) Under pathologic conditions of LRRK2 hyperactivity, increased phosphorylation of RAB GTPases on the AV membrane leads to increased recruitment of JIP4, due to specific binding of JIP4 by RABs in their phosphorylated state. This allows for recruitment and disinhibition of kinesin by JIP4. The presence of active dynein/dynactin and active kinesin on the same organelle results in an unproductive tug-of-war between microtubule-associated motors, leading to non-processive motility and pausing behavior of AVs.

Next, we transiently expressed ARF6^Q67L^-CFP or CFP only in DIV21 PPM1H KO iNeurons (Figure 6A). Similar to overexpression of ARF6 in p.R1441H KI iNeurons, the retrograde fraction was increased, and the stationary fraction was decreased (Figure 6B). Likewise, we observed significant decreases in number of pauses, overall fraction of time paused, number of directional reversals, and Δ run length. We did not observe a change in the pause duration in PPM1H KO iNeurons as a result of ARF6^QL^ expression (Figures 6C–6G). Thus, in two different models of elevated LRRK2-mediated RAB phosphorylation, overexpression of ARF6 partially rescued retrograde axonal transport of AVs. Overall, our findings support a model where increased levels of phosphorylated RABs cause recruitment of additional JIP3/4, which recruits and activates kinesin in the absence of ARF6 regulation. This induces an inappropriately regulated “tug-of-war” that disrupts processive retrograde AV transport. Overexpression of GTP-ARF6 switches surplus JIP3/4 to an activator of dynein/dynactin instead of kinesin and thereby restores AV processivity.

## DISCUSSION

A growing body of evidence links pathogenic LRRK2 mutations to hyperphosphorylation of RAB GTPases, but it remains unclear how phospho-RABs contribute to pathogenesis in PD. Here, we show that increased levels of LRRK2-phosphorylated RAB proteins, resulting from an imbalance in LRRK2 kinase and PPM1H phosphatase activity, disrupt the axonal transport of autophagosomes. Our results further implicate a role for ARF6 in regulating retrograde AV transport (Figure 6H), as expression of GTP-locked ARF6 partially rescued transport deficits induced by either expression of pathogenic forms of LRRK2 or by KO of the opposing phosphatase. We propose that LRRK2-mediated hyperphosphorylation of RAB GTPases induces dysregulation of the opposing motors dynein and kinesin at the AV membrane in a pathogenic “tug-of-war.” Increased recruitment of JIP3/4 in the absence of enhanced levels of ARF6 inappropriately activates kinesin (Figure 6I), which manifests as increased pausing behavior and non-processive motility of axonal AVs.

We found that *LRRK2*-p.R1441H, a kinase-hyperactivating mutation located in the LRRK2 ROC domain, disrupted axonal AV transport to an even greater extent than the p.G2019S mutation we studied in previous work. While both p.G2019S and p.R1441H increased pause frequency and non-processive motility, only p.R1441H increased the average duration of individual pauses and the fraction of AVs remaining stationary. Our data suggest that there is an exacerbation of the severity of pausing behavior in the presence of the more hyperactive mutant LRRK2, to the point where a greater fraction of AVs appears to be fully stationary during the confines of a 5-min live-imaging recording. Combined, the overall fraction of time that AVs spent paused in p.R1441H KI neurons exceeded the effect of p.G2019S by more than 60%. Considering that p.R1441 hotspot mutations cause significantly higher levels of kinase hyperactivity than p.G2019S, our data indicate that the magnitude of AV transport deficits may scale with the level of RAB hyperphosphorylation.

LRRK2 hyperactivity selectively affects axonal cargos, as autophagosome motility is significantly altered by either hyperactivating mutations in LRRK2 or by loss of the countering phosphatase, but no changes were observed in mitochondrial transport or in lysosome trafficking.^10^ We hypothesize that AV motility is specifically disrupted because it is regulated by one or more of the RABs that are LRRK2 substrates. In contrast, there is no known role for RAB binding in the mechanism of axonal trafficking of healthy mitochondria, which is controlled by a complex containing the outer mitochondrial membrane protein Miro, the motor adaptor TRAK, and the motor proteins kinesin-1 and dynein/dynactin.^34^ It is important to note that our live-imaging experiments were all performed at basal conditions, in the absence of pharmacologically induced mitochondrial damage. It has previously been reported that in the presence of high levels of prolonged mitochondrial stress, p.G2019S KI may alter clearance of axonal mitochondria by impairing degradation of Miro and thereby preventing adequate arrest of motile mitochondria.^39^ Further investigation is needed to determine whether such an effect is shared by other pathogenic mutants and whether it can be detected at physiologically relevant levels of mitochondrial damage.

In PPM1H KO iNeurons, the absence of a key phosphatase opposing LRRK2 activity causes increased levels of pRABs in the setting of LRRK2^WT^ (Figure S4B). In a recent study, loss of PPM1H was found to phenocopy the effects of hyperactive LRRK2 on pRAB-dependent ciliation in mouse cholinergic neurons and astrocytes.^32^ Here, we found that loss of PPM1H caused marked deficits of retrograde AV transport in iNeurons, phenocopying the effect of hyperactive LRRK2 on organelle motility. Defective transport was rescued by treatment with MLi-2, confirming that the alterations in AV transport induced by loss of PPM1H are dependent on LRRK2-mediated RAB phosphorylation. We note that the AV transport phenotype observed in PPM1H KO neurons more closely resembled the transport deficits seen in p.R1441H KI (this study) rather than the more limited disruption seen previously in p.G2019S KI neurons. This is likely due to the potent increase in levels of pRABs that we and others observed upon PPM1H KO.^32^ Overall, our data imply an important balance between LRRK2 and PPM1H activity in the regulation of AV transport.

Further, in both PPM1H KO and p.R1441H KI iNeurons, we found that overexpression of the active, GTP-locked mutant ARF6^QL^ ameliorates defects in AV transport. GTP-ARF6 favors the interaction of JIP3/4 with dynein/dynactin rather than kinesin^23^ and is thus well positioned as a regulatory switch that promotes uncontested retrograde AV transport. Recent work supports a role for ARF6 in the regulation of AV transport in neurons, as overexpression of the inactive, GDP-locked mutant ARF6^T27N^ impairs retrograde AV transport along the axon.^38^ We propose that hyperphosphorylated RABs disrupt AV transport by recruiting additional JIP3/4 to the AV membrane, leading to erroneous activation of kinesin and causing an unregulated “tug-of-war” between anterograde and retrograde motor proteins, while overexpressed GTP-ARF6 acts as a switch to favor the JIP3/4-dependent activation of dynein/dynactin, thereby restoring highly processive retrograde motility.

How may defects in axonal AV transport be tied to neurodegeneration in PD? The processive transport of AVs is tightly linked to their function as degradative organelles.^21,22^ Disruption of AV transport induces defective AV acidification and impaired autophagosomal cargo degradation across multiple models of neurodegenerative disease.^10,20,37^ Recent proteomic work has identified nucleoid-enriched mitochondrial fragments as a main cargo of basal autophagy in neurons.^40^ Thus, defects in neuronal autophagy may lead to the aberrant accumulation of mitochondrial DNA with the potential to stimulate pro-inflammatory pathways^41^ and thus contribute to the inflammation characteristic of PD progression.^42^ The degradation of other cargos may also be affected, including the synaptically enriched protein alpha-synuclein, a key protein in PD pathology and an established substrate of autophagy.^43–45^ Consistent with this possibility, exacerbated alpha-synuclein aggregation has been linked to expression of hyperactive LRRK2.^24,46–49^ Ongoing work to examine the degradation of alpha-synuclein in cellular and animal models of PD should shed further light on this question. Of additional interest, we observe low levels of AV pausing in WT conditions, consistent with baseline WT levels of LRRK2 activity. Whether AV pausing has any physiologic function is yet to be explored.

In summary, our data directly link LRRK2-mediated hyperphosphorylation of RAB GTPases to disruption of retrograde AV transport. Mechanistically, we propose that an imbalance between ARF6 and JIP3/4, recruited to the AV membrane by phospho-RABs, disrupts AV transport by inducing an unproductive “tug-of-war” between anterograde and retrograde motor proteins. Our results indicate that the severity of AV transport deficits may scale with the level of LRRK2 hyperactivity, and our data reveal an important balance between LRRK2 kinase activity and the LRRK2-opposing phosphatase PPM1H in regulating AV transport. Together, our work provides insights on how multiple potential therapeutic targets interact in a pathological mechanism that disrupts axonal autophagy, a homeostatic pathway essential for neuronal health and function.

### Limitations of the study

While our study robustly links LRRK2-mediated RAB phosphorylation to impairment of AV transport, we have not yet identified the specific pRAB protein(s) mediating this phenotype. The most likely RABs involved in the LRRK2-dependent impairment of AV transport are RAB10 and/or RAB35, as these RABs are dephosphory-lated by PPM1H^12^ and have been implicated in the recruitment of JIP3/4 to organelles.^7,8^ Proteomic and western blot analyses indicate that RAB10 and RAB35 as well as PPM1H and LRRK2 are all associated with the outer membrane of AVs isolated from brain lysates.^10,40^ However, we cannot exclude the possibility that an as yet unidentified pRAB is involved. Furthermore, it is likely that other phosphatases aside from PPM1H contribute to RAB phosphorylation levels, as suggested by the observed decrease in pRAB levels upon MLi-2 treatment in PPM1H KO neurons. In addition, we acknowledge that more work will be necessary to uncover how defects in AV transport are tied to neurodegeneration in PD.

## STAR★METHODS

### RESOURCE AVAILABILITY

#### Lead contact

Further information and requests for resources and reagents should be directed to and will be fulfilled by the lead contact, Erika L. F. Holzbaur (holzbaur@pennmedicine.edu).

#### Materials availability

Unique reagents generated in this study are available from the Lead Contact with a completed Materials Transfer Agreement. Plasmids generated in this study have been deposited to Addgene (identifier numbers listed in key resources table). iPSC lines generated in this study have been deposited to Cellosaurus (RRID listed in the key resources table).

#### Data and code availability

- Primary data that is presented in this study has been deposited in a Zenodo repository and are publicly available as of the date of publication. These can be accessed using the Digital Object Identifier Zenodo: https://doi.org/10.5281/zenodo.7864259.
- The custom MATLAB scripts used in this study to manually track kymographs (KymoSuite) are available at https://github.com/jnirschl/kinesin-3_guedes-dias_2018/tree/master/kymoSuite (Zenodo: https://zenodo.org/record/2530934). Protocols have been deposited to Protocols.io; links are found in corresponding sections of Method Details.
- Any additional information required to reanalyze the data reported in this paper is available from the lead contact upon request.

### EXPERIMENTAL MODEL AND SUBJECT DETAILS

#### Primary neuron culture

All experiments were performed following protocols approved by the Institutional Animal Care and Use Committee at the University of Pennsylvania. *Lrrk2*-p.G2019S KI mice (model #13940) and B6NTac mice (model #B6) were obtained from Taconic, Cambridge City, Indiana production site. Mouse cortices were dissected from homozygous WT or *Lrrk2*-p.G2019S embryos of either sex at day 15.5. Cortical neurons were isolated by digestion with 0.25% Trypsin and trituration through a small-bore serological pipette. Neurons were plated on 35 mm glass-bottom imaging dishes (P35G-1.5-20-C; MatTek) in Attachment Media (MEM supplemented with 10% horse serum, 33 mM D-glucose and 1 mM sodium pyruvate). After 5 hours, Attachment Media was replaced with Maintenance Media (Neurobasal [GIBCO] supplemented with 2% B-27 [GIBCO], 33 mM D-glucose [Sigma], 2 mM GlutaMAX [GIBCO], 100 U/mL penicillin and 100 mg/mL streptomycin [Sigma]). AraC (1 μM) was added the day after plating to prevent glia cell proliferation. 40% of the media was replaced with fresh Maintenance Media twice per week. Transfections of DIV6 mouse cortical neurons were performed 16-24 hours before imaging using Lipofectamine 2000 Transfection Reagent (Thermo Fisher) and 0.9 μg total plasmid DNA. Published protocol can be found on Protocols.io (https://doi.org/10.17504/protocols.io.81wgby723vpk/v1).

#### i^3^Neuron differentiation

Pre-i^3^Neuron iPSCs (human iPSCs with an integrated doxycycline-inducible mNGN2 transgene in the AAVS1 safe-harbor locus) were a gift from M. Ward (National Institutes of Health, Maryland) and have been described previously.^10,36,52^ Cytogenetic analysis of G-banded metaphases cells showed a normal male karyotype (Cell Line Genetics). Mycoplasma testing was negative. Pre-i^3^N iPSCs were cultured on plates coated with Growth Factor Reduced Matrigel (Corning) and fed daily with Essential 8 media (Thermo Fisher). Differentiation of iPSCs into i^3^Neurons was performed using an established protocol.^52^ In brief, iPSCs were passaged using Accutase (Sigma) and plated on Matrigel-coated dishes in Induction Media (DMEM/F12 supplemented with 1% N2-supplement [GIBCO], 1% NEAA [GIBCO], and 1% GlutaMAX [GIBCO], and containing 2 μg/mL doxycycline. After 72 hours of doxycycline exposure, i^3^Neurons were dissociated with Accutase and cryo-preserved in liquid N2. Published protocol can be found on Protocols.io (https://doi.org/10.17504/protocols.io.261ge348yl47/v1).

#### Piggybac-mediated iPSC-derived neuron differentiation

KOLF2.1J-background WT and *LRRK2*-p.R1441H KI iPSCs were a gift from B. Skarnes (Jackson Laboratories, Connecticut) as part of the iPSC Neurodegenerative Disease Initiative (iNDI) and have been described previously.^29^ KOLF2.1J-background PPM1H KO iPSCs were generated as described below. Cytogenetic analysis of G-banded metaphases cells showed a normal male karyotype (Cell Line Genetics). Mycoplasma testing was negative. iPSCs were cultured on plates coated with Growth Factor Reduced Matrigel (Corning) and fed daily with Essential 8 media (Thermo Fisher). To stably express doxycycline-inducible hNGN2 using a PiggyBac delivery system, iPSCs were transfected with PB-TO-hNGN2 vector (gift from M. Ward, NIH, Maryland) in a 1:2 ratio (transposase:-vector) using Lipofectamine Stem (Thermo Fisher). After 72 hours, transfected iPSCs were selected for 48 hours with 0.5 μg/mL puromycin (Takara). Induction into neuronal fate with doxycycline and cryopreservation of pre-differentiated neurons was performed as described above (“i^3^Neuron differentiation”). Published protocol can be found on Protocols.io (https://doi.org/10.17504/protocols.io.e6nvwj54dlmk/v1).

#### Culture and transfection of iPSC-derived neurons

Cryo-preserved, pre-differentiated iNeurons (i^3^Neurons or Piggybac-delivered NGN2 neurons) were thawed and plated on live-imaging dishes (MatTek) coated with poly-L-ornithine at a density of 300,000 neurons per dish. For each experimental condition, cells from at least two different batches of induction were used over three or more independent experimental cultures. iPSC-derived neurons were cultured in BrainPhys Neuronal Media (StemCell) supplemented with 2% B-27 (GIBCO), 10 ng/mL BDNF (PeproTech), 10 ng/mL NT-3 (PeproTech), and 1 μg/mL laminin (Corning). 40% of the media was replaced with fresh media twice per week. For Piggybac-delivered NGN2 neurons, 10 μM 5-Fluoro-2′-deoxyuridine and 10 μM uridine were included at the time of plating to prevent survival of mitotic cells. These drugs were removed 24 hours after plating. Live imaging experiments were performed 21 days after thawing pre-differentiated iPSC-derived neurons (DIV21). On DIV18, iPSC-derived neurons were transfected with Lipofectamine Stem (Thermo Fisher) and 1-2.5 μg total plasmid DNA. Somal pT73 RAB10 immunostaining (Figure S2C) was performed at DIV7. Published protocol can be found on Protocols.io (https://doi.org/10.17504/protocols.io.x54v9dj4zg3e/v1).

### METHOD DETAILS

#### Plasmids

Plasmids used include PGK EGFP-LC3 (Addgene plasmid #200427; subcloned from PGK mCherry-LC3B, gift from Michael Ward, National Institutes of Health, Maryland), PGK 4xMito-mEmerald (Addgene plasmid #200430; subcloned from 4xMito-mScarlet-I, Addgene plasmid #98818), PGK ARF6^Q67L^-CFP (Addgene plasmid #200428; subcloned from CMVARF6^Q67L^-CFP, Addgene plasmid #11387), PGK CFP (Addgene plasmid #200429; subcloned from CMV ARF6^Q67L^-CFP, Addgene plasmid #11387), CMV mScarlet-LC3B (Addgene plasmid #200431; subcloned from EGFP-LC3B, gift from T. Yoshimori, Osaka University, Japan, with mScarlet from Addgene plasmid #85054), PGK mScarlet-LC3B (Addgene plasmid #200083; subcloned from CMV mScarlet-LC3B, Addgene plasmid #200431), CMV EGFP-PPM1H-WT (#DU62939, MRC PPU reagents and services, University of Dundee, Scotland), PGK EGFP-PPM1H-WT (Addgene plasmid #200084, subcloned from #DU62939, MRC PPU reagents and services, University of Dundee, Scotland), CMV EGFP-PPM1H-H153D (subcloned from pLVX HA PPM1H H153D #DU66215, acquired from MRC PPU reagents and services, University of Dundee, Scotland), CMV FLAG-LRRK2-R1441G (#DU13077, MRC PPU reagents and services, University of Dundee, Scotland), PB-TO-hNGN2 (gift from iPSC Neurodegenerative Disease Initiative (iNDI) & Michael Ward, Addgene plasmid #172115), and piggyBac^™^ transposase vector (available from Transposagen).

#### Generation of PPM1H KO iPSCs

For CRISPR/Cas9 gene editing, KOLF2.1J iPSCs were cultured on Matrigel coated plates in mTeSR medium (StemCell). 800,000 iPSCs were individualized with Accutase and plated onto one well of a Matrigel-coated 6-well plate. iPSCs were then transfected with synthetic sgRNA (Synthego, Gene knockout kit v2 – PPM1H), recombinant Alt-R HiFi Cas9 Nuclease V3 (IDT), and a plasmid encoding for GFP using Lipofectamine Stem Transfection Reagent (Thermo Fisher) following the manufacturers’ instructions (12 μL of 1 μM HiFi Cas9 + 12 μL of 1 μM sgRNA in 76 μL OptiMEM combined with 4 μL Lipofectamine Stem in 100 μL OptiMEM). On the following day, iPSCs were split with Accutase and plated in 6 wells of a 6-well plate. 72 hours after transfection, iPSCs were sorted through FACS and 10,000 GFP+ cells were plated on a Matrigel-coated 10 cm dish. Cells were grown for 9 days, then individual colonies were picked. Successful editing was confirmed by Western blot. Karyotype analysis (Cell Line Genetics) demonstrated a normal karyotype of the clone used in this study. Published protocol can be found on Protocols.io (https://doi.org/10.17504/protocols.io.36wgqj48yvk5/v1).

#### Live-cell imaging

Primary mouse cortical neurons were imaged on DIV7 in low fluorescence Hibernate E medium (Brain Bits) supplemented with 2% B27 and 2 mM GlutaMAX. iNeurons were imaged on DIV21 in low fluorescence Hibernate A medium (Brain Bits) supplemented with 2% B27,10 ng/mL BDNF and 10 ng/mL NT-3. Neurons were imaged in an environmental chamber at 37°C. Recordings of mScarlet-LC3 vesicles in mouse cortical neurons as well as EGFP-LC3 vesicles and mitochondria in WT vs. LRRK2-R1441H iNeurons were acquired on a PerkinElmer UltraView Vox Spinning Disk Confocal system with a Nikon Eclipse Ti inverted microscope, using a Plan Apochromat 60x 1.40 NAoil immersion objective and a Hamamatsu EMCCD C9100-50 camera controlled by Volocity software. Following a scheduled microscope upgrade, the last replicate of the live imaging experiment investigating mitochondrial transport in *LRRK2*-p.R1441H KI iNeurons and all replicates of the experiment investigating the effect of ARF6^Q67L^-CFP overexpression in *LRRK2*-p.R1441H iNeurons were instead performed using a Hamamatsu ORCA-Fusion C14440-20UP camera controlled by VisiView software. Experiments investigating EGFP-LC3 transport in PPM1H KO iNeurons were performed at the Live-Cell Imaging Facility of the Max Planck Institute for Multidisciplinary Sciences, Goettingen, on a Visitron CSU-W1 Spinning Disk Confocal system with a Nikon Ti2 inverted microscope using a Plan Apochromat 60x 1.40 NA oil immersion objective and a Prime BSI sCMOS camera controlled by VisiView software. Axons were identified based on morphological parameters.^36,53^ All time lapse recordings were acquired in the mid-axon (> 300 μm from the soma and >100 μm from the distal axon terminal) at a frame rate of 1 frame/sec for 5 minutes. Published protocol can be found on Protocols.io (https://doi.org/10.17504/protocols.io.rm7vzb3r4vx1/v1).

#### Immunoblotting

Neurons or MEFs were washed twice with ice cold PBS and lysed with RIPA buffer (50 mM Tris-HCl, 150 mM NaCl, 0.1% Triton X-100, 0.5%deoxycholate, 0.1% SDS, 2x Halt Protease and Phosphatase inhibitor, 2mg/mL microcystin-LR). Samples were centrifuged for 10 min at 17,000 g, and protein concentration of the supernatant was determined by BCA assay. Proteins were resolved on 8% (LRRK2) - 15% (PPM1H or RAB proteins) acrylamide gels. Proteins were transferred to Immobilon-FL PVDF membranes (Milli-pore) using a wet blot transfer system. Membranes were then stained for total protein using LI-COR Revert 700 Total Protein Stain. Following imaging of total protein stain, membranes were de-stained and blocked for 5 minutes with Bio-Rad EveryBlot blocking buffer. For PPM1H and pan-specific phosphothreonine RAB blots, membranes were incubated with primary antibody diluted in EveryBlot for 90 minutes at room temperature. For pT73 RAB10 and total RAB10 blots (Figures S1–S3), membranes were incubated with primary antibody diluted in EveryBlot at 4°C overnight. After three washes with TBS (50 mM Tris-HCl [pH 7.4], 274 mM NaCl, 9 mM KCl) with 0.1% Tween-20, membranes were incubated with secondary antibodies diluted in EveryBlot with 0.01% SDS for 1 hr at RT. Following three more washes with TBS with 0.1% Tween-20, membranes were imaged using an Odyssey CLx Infrared Imaging System (LI-COR). Western blots were analyzed with Image Studio Software (LI-COR). Published protocol can be found on Protocols.io (https://doi.org/10.17504/protocols.io.5jyl8j5zrg2w/v1).

#### Immunostaining

Human iNeurons were fixed in Bouin’s solution (Sigma) and BrainPhys media (1:1) for 30 minutes, washed five times with PBS, and permeabilized for 8 min at −20°C using ice-cold methanol. Cells were then washed three times with PBS and blocked for 1 h with 5% goat serum and 1% BSA in PBS. Neurons were then incubated in primary antibody (ab241060, 1:100) diluted in blocking solution at room temperature for 1 h, washed three times with PBS, and incubated in secondary antibody (1:1000) diluted in blocking solution for 1 h at room temperature. After three washes with PBS, coverslips were mounted in ProLong Gold Antifade Mountant (Thermo Fisher). Images were acquired as z stacks at 200 nm step-size using a spinning disk confocal setup as described above. Published protocol can be found on Protocols.io (https://doi.org/10.17504/protocols.io.e6nvwj4kdlmk/v1).

### QUANTIFICATION AND STATISTICAL ANALYSIS

Kymographs of axonal autophagosomes and mitochondria were generated using the Multiple Kymograph plugin (FIJI). Line width was set to 5 pixels. Tracks were traced manually using a custom MATLAB GUI (KymoSuite). Motile autophagosomes and mitochondria were scored as anterograde (net displacement >10 μm in the anterograde direction within the 5-minute time lapse duration), retrograde (net displacement >10 μm in the retrograde direction), or bi-directional (net displacement <10 μm in either direction but total displacement >10 μm). Autophagosomes and mitochondria were scored as stationary if net and total displacement were <10 μm. A pause was defined as a single or consecutive instantaneous velocity value of <0.083 μm/s. Bi-directional organelles were included in quantification of pause number, pause duration, directional reversals, and Δ run length, but stationary organelles were excluded. Quantification of the fraction of time paused included all (motile and stationary) autophagosomes. For quantification of Δ run length, the net run length of each vesicle was subtracted from its total run length (Figure S2E). All analyses were performed by a blinded investigator.

Figure legends contain descriptions of the statistical test(s) used, specific p values, sample size, and dispersion/precision measurements. For directionality analysis for both AV and mitochondrial transport experiments, two-way ANOVA analysis with Sidak’s test for multiple comparisons was performed using GraphPad Prism 9. For other transport parameters, RStudio version 2021.9.2.382 was used to perform either a linear mixed effects model (LME; R package “nlme”) or a generalized linear mixed model (GLMM; R package “lme4”). The genotype (or, in MLi-2 experiments, the treatment condition) was treated as the fixed effect. The independent experiment/culture and the neuron being recorded from were treated as nested random effects, with the neuron nested within the experiment. Specific models used were chosen based on distribution of each dataset and diagnostic residual plots. In detail, the following models were used: for fraction of time paused, GLMM (binomial family, with the “weights” argument for total time); for pause number, GLMM (Poisson family); for pause duration, GLMM (gamma family, log link function); for reversals, GLMM (Poisson family); for Δ run length, GLMM (gamma family, log link function, with transformation to remove zero values with added constants); for mito density, LME; for mito length, LME. In situations where gamma family GLMMs failed to converge (Figures 1F, 3D, 4G, 6D, 6F, and S3F), adaptive Gauss-Hermite quadrature (nAGQ=0) was used instead of the default Laplace approximation (nAGQ=1). For all quantifications, at least three independent experiments were analyzed.

## Supplementary Material

1

2

3

4

5

6

7

## Figures and Tables

**Table:** KEY RESOURCES TABLE

REAGENT or RESOURCE	SOURCE	IDENTIFIER
Antibodies		
Anti-RAB10, Rabbit Monoclonal	Abcam	Cat# ab237703; RRID:AB_2884879
Anti-RAB10 (phospho T73), Rabbit Monoclonal (used for Western blot)	Abcam	Cat# ab230261; RRID:AB_2811274
Anti-RAB10 (phospho T73), Rabbit Monoclonal (used for immunofluorescence)	Abcam	Cat# ab241060; RRID:AB_2884876
Anti-RAB8A (phospho T72), Rabbit Monoclonal	Abcam	Cat# ab230260; RRID:AB_2814988
Anti-PPM1H, Sheep Polyclonal	MRC PPU reagents and services, University of Dundee, Scotland	#DA018, 3^rd^ bleed; RRID:AB_2923281
Anti-Rabbit IgG-IRDye 800CW, Donkey Polyclonal	Li-COR Biosciences	Cat# 926-32213; RRID:AB_621848
Anti-Rabbit IgG-IRDye 680RD, Donkey Polyclonal	Li-COR Biosciences	Cat# 926-68073; RRID:AB_10954442
Anti-Mouse IgG-IRDye 800CW, Donkey Polyclonal	Li-COR Biosciences	Cat# 926-32212; RRID:AB_621847
Anti-Sheep Alexa Fluor 680, Donkey Polyclonal	Thermo Fisher	Cat# A21102; RRID:AB_10374469
Anti-Rabbit IgG Alexa Fluor 488, Goat Polyclonal	Thermo Fisher	Cat# A-11034; RRID:AB_2576217
Chemicals, peptides, and recombinant proteins		
MLi-2	Tocris	Cat# 5756
DMSO	Sigma-Aldrich	Cat# D2650
PLL (mol wt 70,000–150,000)	Sigma-Aldrich	Cat# P1274
HBSS (10x)	Thermo Fisher	Cat# 14185-052
1M HEPES	Thermo Fisher	Cat# 15630-080
2.5% Trypsin	Thermo Fisher	Cat# 15090-046
Minimum essential medium (MEM)	Thermo Fisher	Cat# 11095-072
Horse serum (heat inactivated)	Thermo Fisher	Cat# 16050-122
Sodium pyruvate	Corning	Cat# 36017004
D-Glucose solution 45%	Sigma-Aldrich	Cat# G8769
GlutaMAX	Thermo Fisher	Cat# 35050061
B27 Supplement	Thermo Fisher	Cat# 17504-044
Neurobasal Medium	Thermo Fisher	Cat# 21103-049
Penicillin-Streptomycin	Thermo Fisher	Cat# 15140-122
AraC	Sigma-Aldrich	Cat# C6645
Lipofectamine 2000 Transfection Reagent	Thermo Fisher	Cat# 11668019
Matrigel Growth Factor Reduced	Corning	Cat# 354230
Essential 8 Medium	Thermo Fisher	Cat# A1517001
mTeSR Medium	Stemcell Technologies	Cat# 85850
Alt-R HiFi Cas9 Nuclease V3	IDT	Cat# 1081060
ReLeSR	Stemcell Technologies	Cat# 05872
Accutase	Sigma-Aldrich	Cat# A6964
ROCK Inhibitor Y-27632	Selleckchem	Cat# S1049
Tet System Approved FBS	Takara	Cat# 631107
Knockout Serum Replacement	Thermo Fisher	Cat# 10828010
DMEM/F-12, HEPES	Thermo Fisher	Cat# 11330032
N2 Supplement	Thermo Fisher	Cat# 17502048
Non-essential Amino Acids (NEAA)	Thermo Fisher	Cat# 11140050
Doxycycline	Sigma-Aldrich	Cat# D9891
Poly-L-Ornithine	Sigma-Aldrich	Cat# P3655
BrainPhys Neuronal Medium	Stemcell Technologies	Cat# 05790
Laminin	Corning	Cat# 354232
BDNF	PeproTech	Cat# 450-02
NT-3	PeproTech	Cat# 450-03
Lipofectamine Stem Transfection Reagent	Thermo Fisher	Cat# STEM00003
Microcystin-LR	Sigma-Aldrich	475815
Halt Protease and Phosphatase Inhibitor Cocktail	Thermo Fisher	78442
5-Fluoro-2′-deoxyuridine	Sigma-Aldrich	F0503
Uridine	Sigma-Aldrich	U3003
Bouin’s solution	Sigma-Aldrich	HT10132
Critical commercial assays		
BCA Protein Assay Kit	Thermo Fisher	Cat# 23225
Plasmid Maxi Kit	QIAGEN	Cat# 12163
Experimental models: Cell lines		
Human: NGN2 iPSCs	M. Ward (National Institutes of Health)^50^	RRID: CVCL_C7XJ
Human: LRRK2-G2019S NGN2 iPSCs (heterozygous)	(Boecker et al., 2021)^10^	RRID: CVCL_C7XK
Mouse: LRRK2-G2019S knock-in MEFs	(Boecker et al., 2021)^10^	RRID: CVCL_C7XL
Mouse: WT MEFs	MRC PPU reagents and services, University of Dundee, Scotland	RRID: CVCL_L690
Mouse: LRRK2-R1441C	MRC PPU reagents and services,	N/A
knock-in MEFs	University of Dundee, Scotland	
Human: KOLF2.1J WT iPSCs	B. Skarnes (Jackson Laboratories, Connecticut)	RRID: CVCL_B5P3
Human: KOLF2.1J LRRK2-R1441H iPSCs	B. Skarnes (Jackson Laboratories, Connecticut)	N/A
Human: KOLF2.1J PPM1H KO iPSCs	This paper	RRID: CVCL_C7TY
Experimental models: Organisms/strains		
Mouse: LRRK2-G2019S knock-in	Taconic	Model #13940
Mouse: C57BL/6NTac WT	Taconic	Model #B6; RRID: IMSR_TAC:b6
Oligonucleotides		
Gene knockout kit v2 – PPM1H (human)	Synthego	N/A
Recombinant DNA		
Plasmid: PGK EGFP-LC3B	Modified from PGK mCherry-LC3B (gift from Michael Ward, NIH)	Addgene plasmid #200427
Plasmid: PGK ARF6^Q67L^-CFP	This paper	Addgene plasmid #200428 (modified from Addgene plasmid #11387)
Plasmid: PGK CFP	This paper	Addgene plasmid #200429 (modified from Addgene plasmid #11387)
Plasmid: PGK 4xMito-mEmerald	This paper	Addgene plasmid #200430 (modified from Addgene plasmid #98818)
Plasmid: CMV mScarlet-LC3B	Modified from EGFP-LC3B (gift from T. Yoshimori, Osaka University, Japan)	Addgene plasmid #200431
Plasmid: PGK mScarlet-LC3B	This paper	Addgene plasmid #200083 (modified from Addgene plasmid #200431)
Plasmid: CMV EGFP-PPM1H-WT	MRC PPU reagents and services, University of Dundee, Scotland	#DU62939
Plasmid: PGK EGFP-PPM1H-WT	This paper	Addgene plasmid #200084 (modified from #DU62939)
Plasmid: CMV EGFP-PPM1H-H153D	This paper	Addgene plasmid #200432 (modified from MRC PPU #DU66215)
Plasmid: CMV FLAG-LRRK2-R1441G	MRC PPU reagents and services, University of Dundee, Scotland	#DU13077
Plasmid: PB-TO-hNGN2	Gift from iPSC Neurodegenerative Disease Initiative (iNDI) & Michael Ward	Addgene plasmid #172115
Plasmid: piggyBac^™^ transposase vector	Transposagen	N/A
Software and algorithms		
FIJI (Release 2.9.0)	NIH, USA	http://fiji.sc; RRID:SCR_002285
Prism 9	GraphPad	https://www.graphpad.com/scientific-software/prism/; RRID:SCR_002798
RStudio: Integrated Development for R (2021.09.2 Build 382)	RStudio Team	http://www.rstudio.com/; RRID:SCR_000432
R package: nlme	Pinheiro J, Bates D, R Core Team	http://CRAN.R-project.org/package=nlme; RRID:SCR_015655
R package: lme4	Bates D, Mächler M, Bolker B, Walker S	https://cran.r-project.org/web/packages/lme4/index.html; RRID:SCR_015654
Matlab R2022a	MathWorks	https://www.mathworks.com/products/matlab.html; RRID:SCR_001622
KymoSuite (custom Matlab script)	(Guedes-Dias et al., 2019)^51^	https://github.com/jnirschl/kinesin-3_guedes-dias_2018/tree/master/kymoSuite, Zenodo: https://zenodo.org/record/2530934
Volocity	PerkinElmer	https://www.perkinelmer.com; RRID:SCR_002668
VisiView 5.0.0.24	Visitron	https://www.visitron.de/products/visiviewr-software.html; RRID:SCR_022546
LI-COR Image Studio	LI-COR	https://www.licor.com/bio/image-studio/; RRID:SCR_015795
Adobe Illustrator 2022	Adobe	https://www.adobe.com/products/illustrator.html; RRID:SCR_010279
BioRender	BioRender	https://biorender.com/; RRID:SCR_018361
Deposited data		
Raw images and quantitative results	Zenodo	Zenodo: https://doi.org/10.5281/zenodo.7864259
Other		
35 mm #1.5 glass bottom imaging dishes	MatTek	Cat# P35G-1.5-20-C
ProLong Gold Antifade Mountant	Thermo Fisher	Cat# P36930

## Highlights

- Parkinson’s-associated *LRRK2*-p.R1441H impairs axonal autophagosomal transport
- Knockout of LRRK2-opposing phosphatase PPM1H phenocopies hyperactive LRRK2
- Transport deficits are linked to regulatory imbalance between ARF6 and phospho-RABs
