# Supplementary material for: Regulatory imbalance between LRRK2 kinase, PPM1H phosphatase, and ARF6 GTPase disrupts the axonal transport of autophagosomes
Source: Cell Rep. Author manuscript; Available in PMC 2023 Jun 28. (PMC10304398; doi:10.1016/j.celrep.2023.112448)
Supplement: 1 [file NIHMS1905364-supplement-1.pdf]

Cell Reports, Volume *42*

Supplemental information

Regulatory imbalance between LRRK2 kinase, PPM1H phosphatase, and ARF6 GTPase disrupts the axonal transport of autophagosomes

Dan Dou, Erin M. Smith, Chantell S. Evans, C. Alexander Boecker, and Erika L.F. Holzbaur

Figure S1

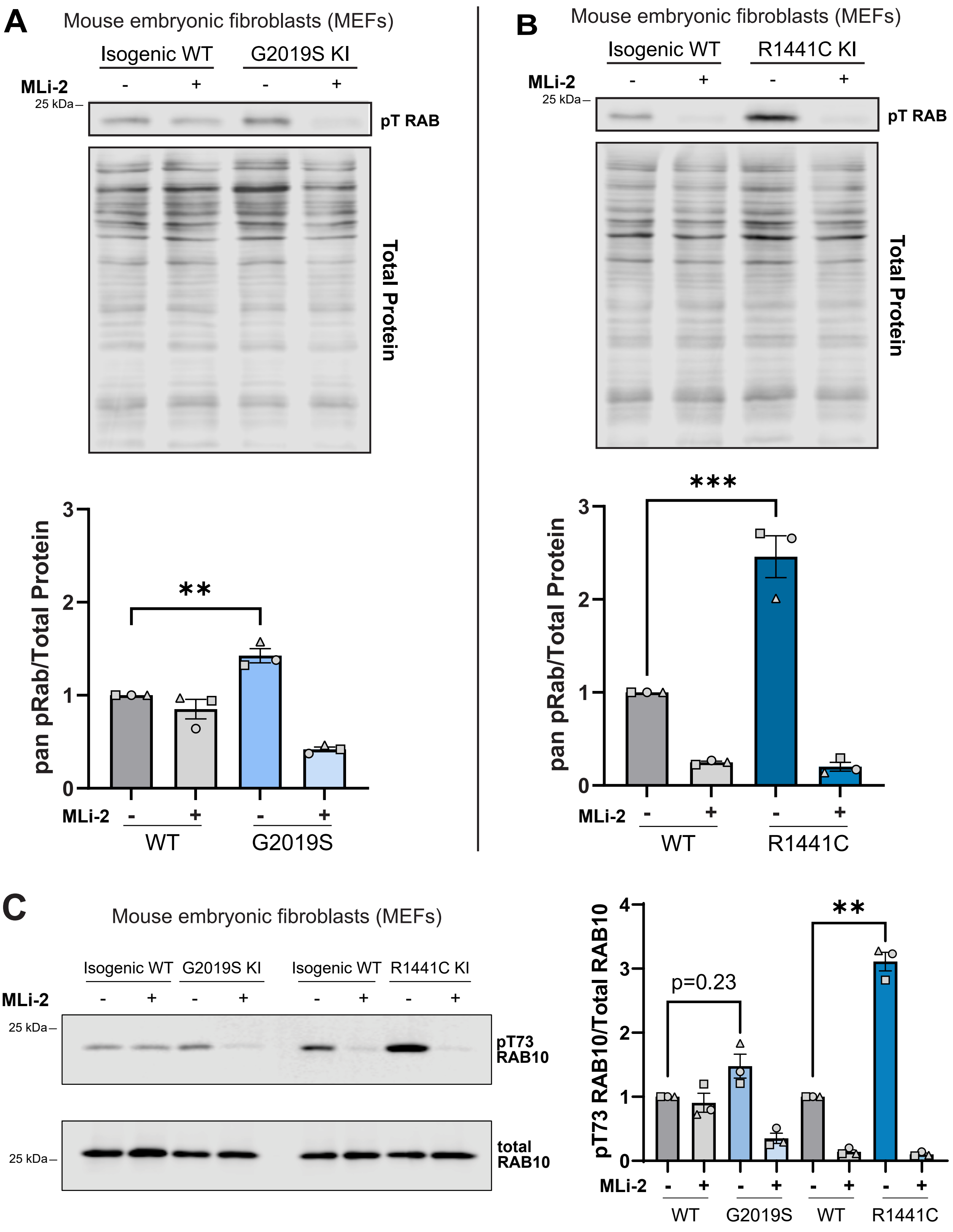

A
Mouse embryonic fibroblasts (MEFs)
Isogenic WT
G2019S KI
MLi-2
25 kDa
pT RAB
Total Protein
pan pRab/Total Protein
**
WT
G2019S
B
Mouse embryonic fibroblasts (MEFs)
Isogenic WT
R1441C KI
MLi-2
25 kDa
pT RAB
Total Protein
pan pRab/Total Protein
***
WT
R1441C
C
Mouse embryonic fibroblasts (MEFs)
Isogenic WT
G2019S KI
Isogenic WT
R1441C KI
MLi-2
25 kDa
pT73 RAB10
total RAB10
pT73 RAB10/Total RAB10
p=0.23
**
WT
G2019S
WT
R1441C

Figure S1. LRRK2 mutant knock-in mouse embryonic fibroblasts (MEFs) have increased levels of LRRK2-phosphorylated RAB proteins. Related to Figure 1. Representative Western blot and quantification of phosphothreonine RABs in (A) *Lrrk2*-p.G2019S KI MEFs and isogenic WT MEFs (mean ± SEM; n=3 biological replicates; **p=0.0073; One-way ANOVA with Tukey's multiple comparisons test) and (B) *Lrrk2*-p.R1441C KI MEFs and isogenic WT MEFs (mean ± SEM; n = 3 biological replicates; ***p=0.0004; One-way ANOVA with Tukey's multiple comparisons test), treated overnight with DMSO or 100 nM MLi-2. Data shown are normalized to total protein and DMSO-treated matched WT condition. (C) Representative Western blot and quantification of pT73 RAB10 and Total RAB10 in *Lrrk2*-p.G2019S KI MEFs, *Lrrk2*-p.R1441C KI MEFs, and isogenic WT MEFs corresponding to each mutant (mean ± SEM; n = 3 biological replicates; **p=0.0094; One-way ANOVA with Tukey's multiple comparisons test), treated overnight with DMSO or 100 nM MLi-2.

Figure S2

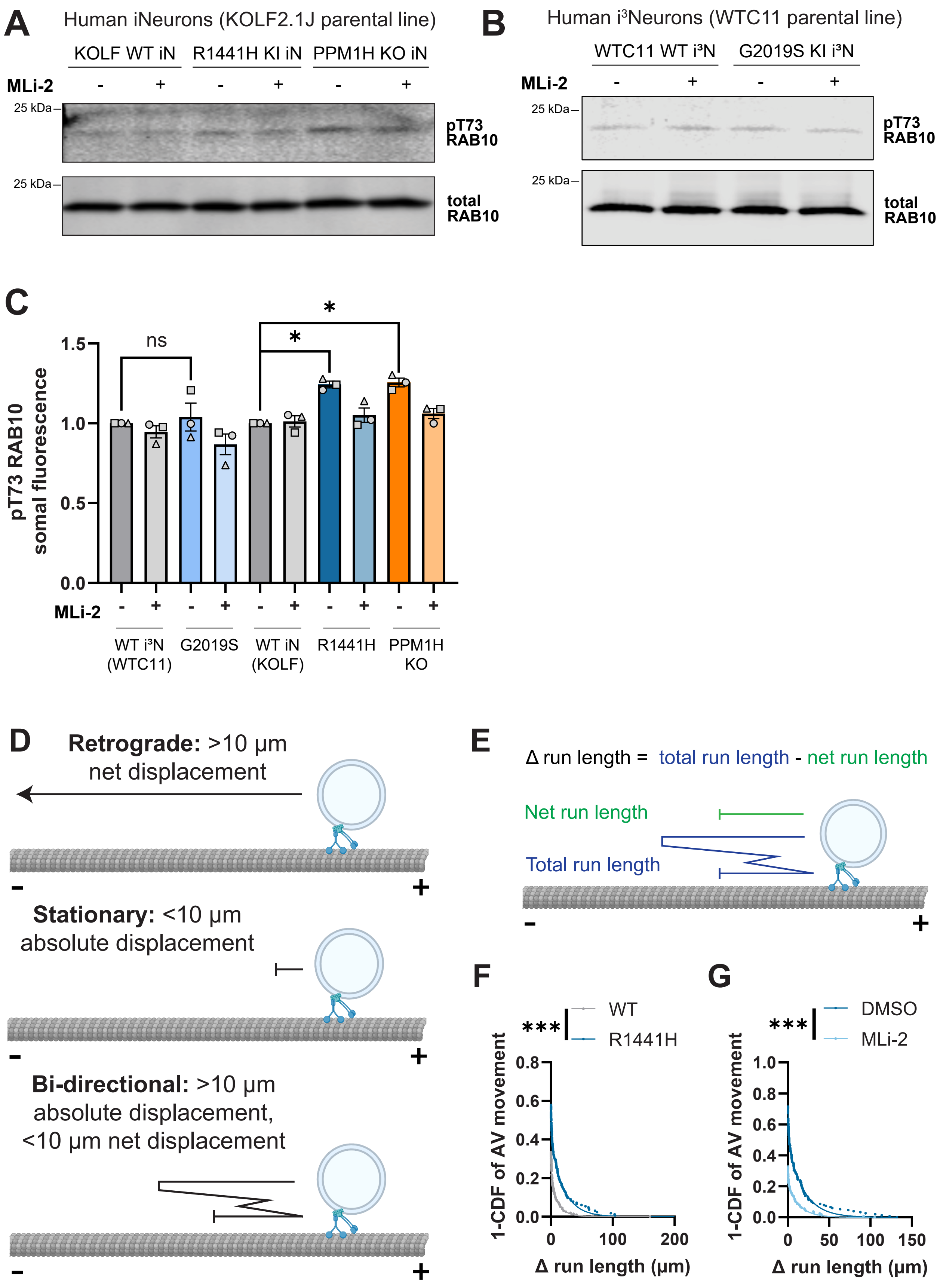

A
Human iNeurons (KOLF2.1J parental line)
KOLF WT iN
R1441H KI iN
PPM1H KO iN
MLi-2
25 kDa
pT73 RAB10
total RAB10
B
Human i3Neurons (WTC11 parental line)
WTC11 WT i3N
G2019S KI i3N
C
ns
pT73 RAB10 somal fluorescence
WT i3N (WTC11)
G2019S
WT iN (KOLF)
R1441H
PPM1H KO
D
Retrograde: >10 μm net displacement
Stationary: <10 μm absolute displacement
Bi-directional: >10 μm absolute displacement, <10 μm net displacement
E
Δ run length = total run length - net run length
Net run length
Total run length
F
WT
R1441H
1-CDF of AV movement
Δ run length (μm)
G
DMSO
MLi-2

Figure S2. *LRRK2*-p.R1441H knock-in iNeurons have decreased processivity of AV transport. Related to Figure 1. (A) pT73 RAB10 Western blot of WT, *LRRK2*-p.R1441H KI, and PPM1H KO iNeurons, treated overnight with DMSO or 200 nM MLi-2. (B) pT73 RAB10 Western blot of WT and p.G2019S KI i^3Neurons, treated overnight with DMSO or 200 nM MLi-2. (C) Quantified somal pT73 RAB10 fluorescence of WT and p.G2019S i^3Neurons and WT, p.R1441H KI, and PPM1H KO iNeurons (mean ± SEM; n = 3 biological replicates; *p<0.0335; One-way ANOVA with Sidak's multiple comparisons test), treated overnight with DMSO or 200 nM MLi-2. (D) Criteria for directionality categorization of AVs in live-imaging experiments. (E) Depiction of Δ run length calculation as a measure of AV transport processivity. (F) Δ run length of AVs in WT and p.R1441H iNeurons (n = 168-200 motile AVs from 31 neurons from 3 independent experiments; ***p<0.001; mixed effects model analysis). (G) Δ run length of AVs in p.R1441H KI iNeurons treated with DMSO or MLi-2 (n = 153-163 motile AVs from 30 neurons from 3 independent experiments; ***p<0.001; mixed effects model analysis). For panels F and G, curve fits were generated using nonlinear regression (two phase decay).

Figure S3

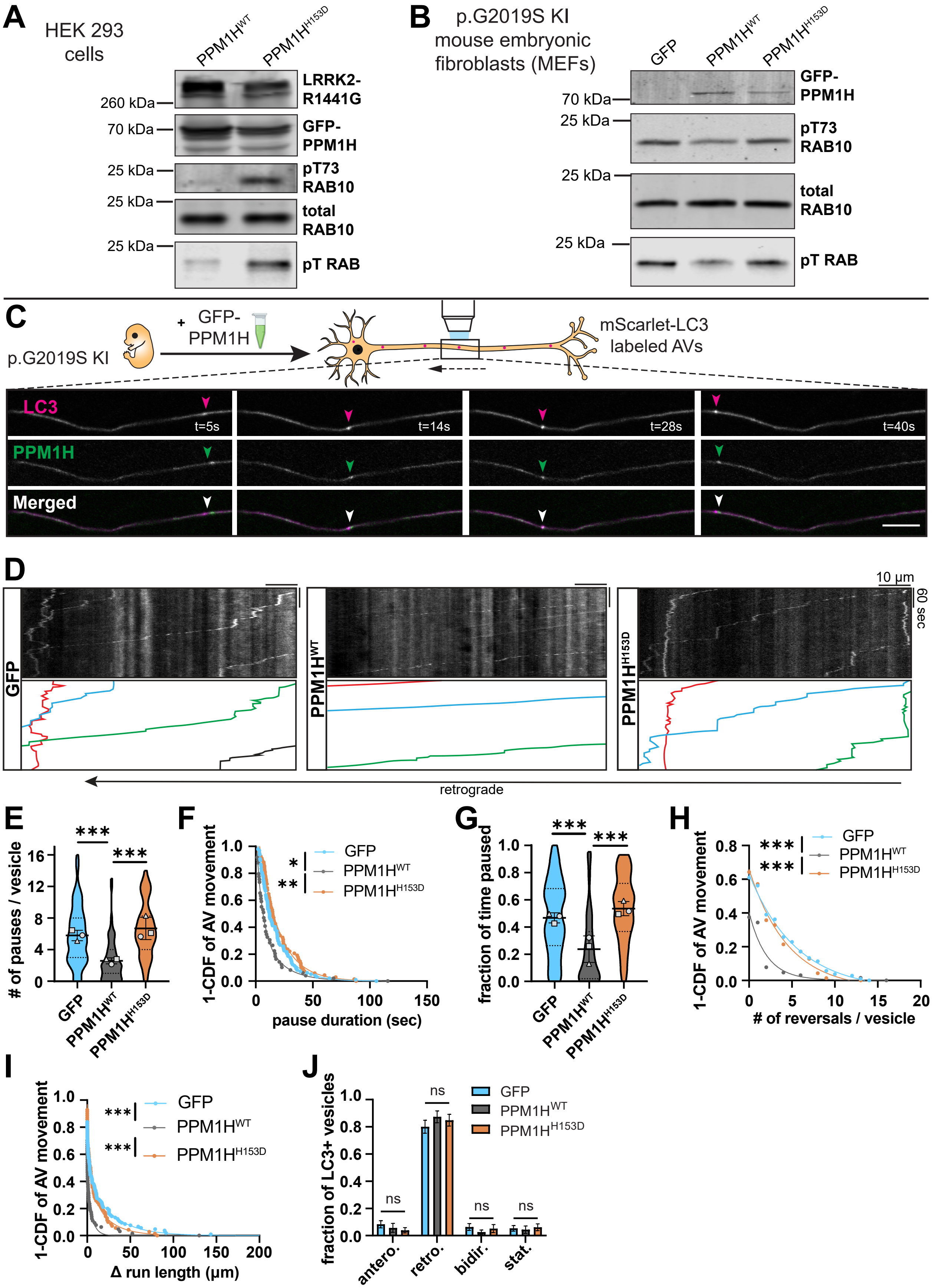
A
HEK 293 cells
PPM1H^WT
PPM1H^H153D
260 kDa
70 kDa
25 kDa
LRRK2-R1441G
GFP-PPM1H
pT73 RAB10
total RAB10
pT RAB
B
p.G2019S KI mouse embryonic fibroblasts (MEFs)
GFP
GFP-PPM1H
C
p.G2019S KI
+ GFP-PPM1H
mScarlet-LC3 labeled AVs
LC3
PPM1H
Merged
t=5s
t=14s
t=28s
t=40s
D
10 µm
60 sec
GFP
PPM1H^WT
PPM1H^H153D
retrograde
E
# of pauses / vesicle
F
1-CDF of AV movement
pause duration (sec)
G
fraction of time paused
H
# of reversals / vesicle
I
Δ run length (µm)
J
fraction of LC3+ vesicles
ns
antero.
retro.
bidir.
stat.

Figure S3. Overexpression of PPM1H rescues AV transport in neurons expressing hyperactive LRRK2. Related to Figure 2. (A) pT73 RAB10 and phosphothreonine RAB Western blot of HEK 293 cells co-expressing LRRK2-R1441G and GFP-PPM1H^{WT} or catalytically inactive GFP-PPM1H^{H153D}. (B) pT73 RAB10 and phosphothreonine RAB Western blot of *Lrrk2*-p.G2019S knock-in MEFs transiently expressing GFP, GFP-PPM1H^{WT}, or GFP-PPM1H^{H153D}. (C) Time lapse images of axonal mScarlet-LC3+ and GFP-PPM1H^{WT}+ vesicles in a p.G2019S KI mouse cortical neuron. Scale bar, 10 µm. (D) Kymographs of axonal mScarlet-LC3+ vesicles in p.G2019S KI mouse cortical neurons co-expressing GFP, GFP-PPM1H^{WT}, or GFP-PPM1H^{H153D}. Example AV traces are highlighted. (E-H) Pause number (E) and pause duration (F) of motile AVs, fraction of time paused (G) of all AVs, and directional reversals (H) of motile AVs in G2019S KI mouse cortical neurons transiently expressing GFP, GFP-PPM1H^{WT}, or GFP-PPM1H^{H153D} (mean ± SD for panel E and G; n = 66-107 motile AVs (E, F, H) and 68-111 total AVs (G) from 21-24 axons from 3 independent experiments; *p=0.01106; **p=0.00144; ***p<0.001; mixed effects model analysis). (I) Δ run length of motile AVs in p.G2019S KI mouse cortical neurons transiently expressing GFP, GFP-PPM1H^{WT}, or GFP-PPM1H^{H153D} (n = 66-107 motile AVs from 21-24 axons from 3 independent experiments; ***p<0.001; mixed effects model analysis). (J) Directionality of AVs in p.G2019S KI mouse cortical neurons transiently expressing GFP, PPM1H^{WT}, or GFP-PPM1H^{H153D}. Antero., anterograde; retro., retrograde; bidir., bi-directional; stat., stationary (mean ± SEM; n = 21-24 neurons from 3 independent experiments; ns, not significant, p>0.2115; two-way ANOVA with Sidak's multiple comparisons test). For panels F, H, and I, curve fits were generated using nonlinear regression (for F and H, two phase decay; for I, three phase decay).

Figure S4

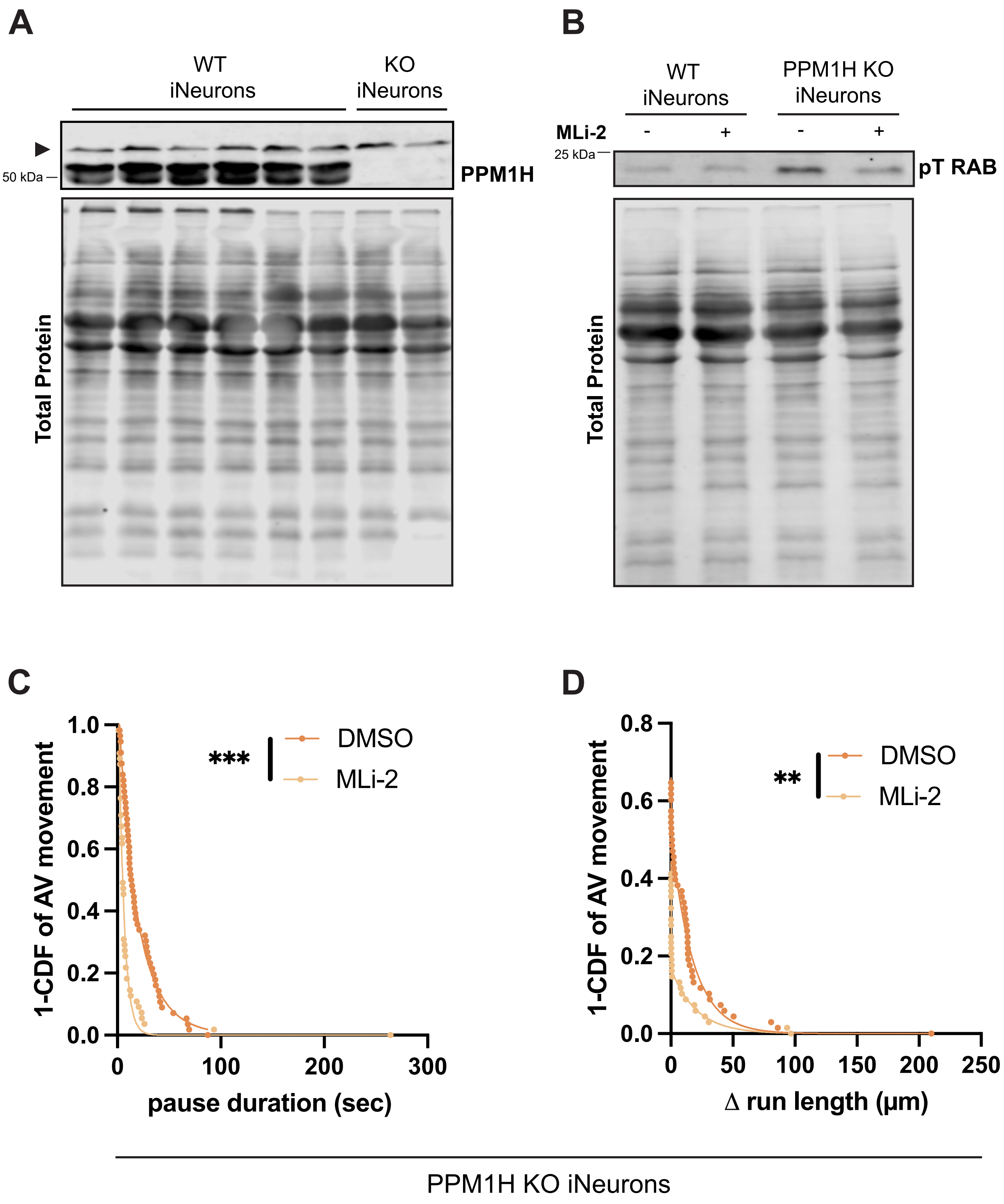

A
WT iNeurons
KO iNeurons
50 kDa
PPM1H
Total Protein
B
WT iNeurons
PPM1H KO iNeurons
MLi-2
-
+
-
+
25 kDa
pT RAB
Total Protein
C
1-CDF of AV movement
***
DMSO
MLi-2
pause duration (sec)
D
1-CDF of AV movement
**
DMSO
MLi-2
Δ run length (μm)
PPM1H KO iNeurons

Figure S4. PPM1H KO iNeurons have increased levels of LRRK2-phosphorylated RAB proteins. Related to Figure 3. (A) PPM1H Western blot of DIV21 WT or PPM1H KO iNeurons. Arrowhead indicates non-specific band. (B) Pan-specific phosphothreonine RAB Western blot of DIV21 WT or PPM1H KO iNeurons treated with DMSO or 100 nM MLi-2 overnight. (C-D) Pause duration (C) and Δ run length (D) of motile AVs in PPM1H KO iNeurons treated with DMSO or MLi-2 for 72 hours (n = 68 motile AVs from 28-30 neurons from 3 independent experiments; **p=0.00348; ***p<0.001; mixed effects model analysis). For panels C and D, curve fits were generated using nonlinear regression (two phase decay).

Figure S5

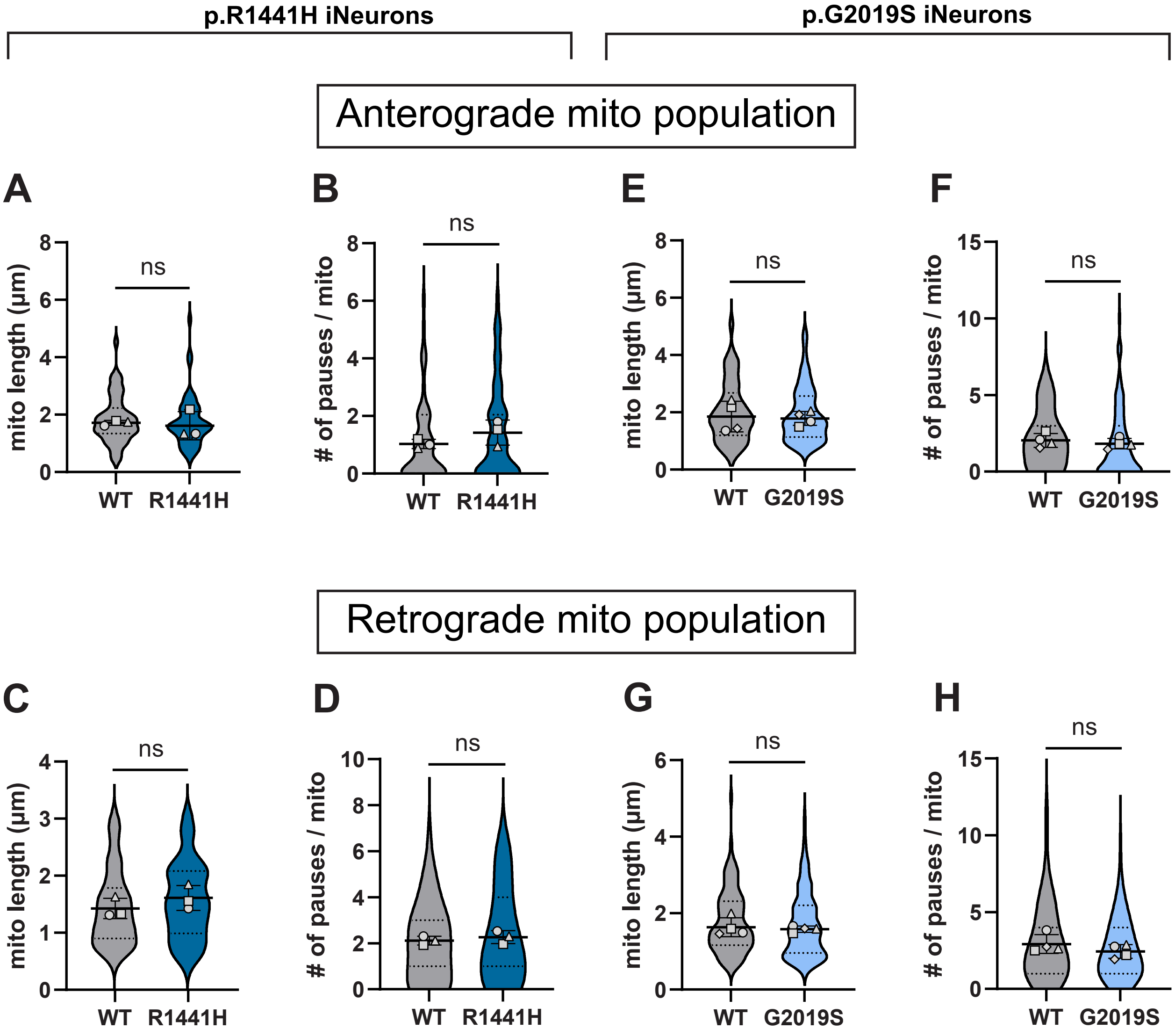

p.R1441H iNeurons
p.G2019S iNeurons
Anterograde mito population
A
B
E
F
mito length (µm)
# of pauses / mito
ns
WT
R1441H
G2019S
Retrograde mito population
C
D
G
H

Figure S5. Pathogenic *LRRK2* mutations do not disrupt anterograde or retrograde subpopulations of axonal mitochondria. Related to Figure 4. (A-B) Average length (A) and pause number (B) of anterograde population of mitochondria in WT and p.R1441H KI iNeurons (mean ± SD; n = 51-54 anterograde mitochondria from 26 neurons from 3 independent experiments; ns, not significant, p>0.527; mixed effects model analysis). (C-D) Average length (C) and pause number (D) of retrograde population of mitochondria in WT and p.R1441H KI iNeurons (mean ± SD; n = 63-96 retrograde mitochondria from 26 neurons from 3 independent experiments; ns, not significant, p>0.1233; mixed effects model analysis). (E-F) Average length (E) and pause number (F) of anterograde population of mitochondria in WT and p.G2019S KI iNeurons (mean ± SD; n = 47-91 anterograde mitochondria from 59-60 neurons from 4 independent experiments; ns, not significant, p>0.5598; mixed effects model analysis). (G-H) Average length (G) and pause number (H) of retrograde population of mitochondria in WT and p.G2019S KI iNeurons (mean ± SD; n = 106-162 retrograde mitochondria from 59-60 neurons from 4 independent experiments; ns, not significant, p>0.103; mixed effects model analysis). Scatter plot points indicate the means of 3-4 independent experiments.

Figure S6

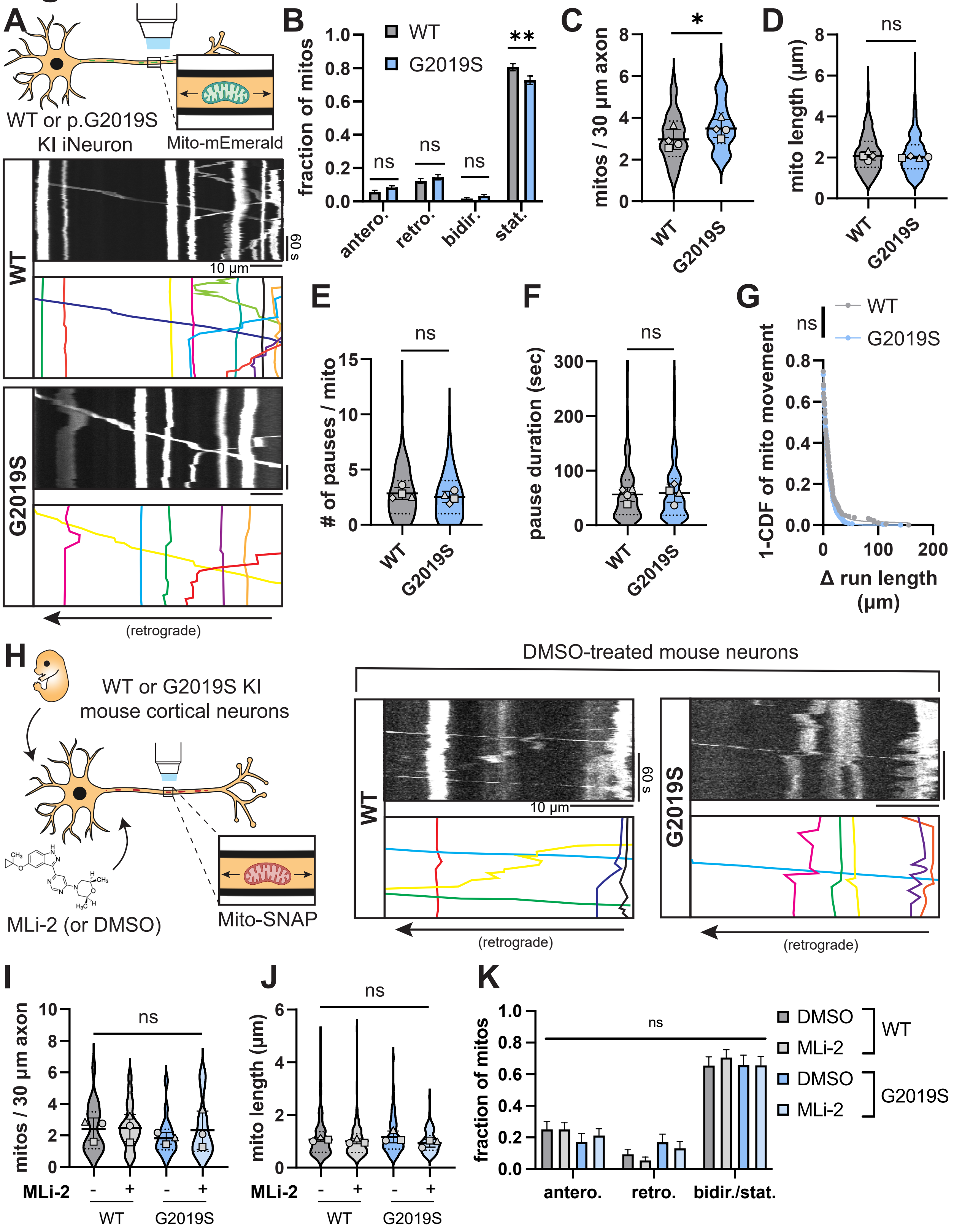
A
WT or p.G2019S
KI iNeuron
Mito-mEmerald
WT
G2019S
60 s
10 μm
(retrograde)
B
fraction of mitos
WT
G2019S
ns
ns
ns
**
antero.
retro.
bidir.
stat.
C
mitos / 30 μm axon
*
WT
G2019S
D
mito length (μm)
ns
E
# of pauses / mito
ns
F
pause duration (sec)
ns
G
1-CDF of mito movement
ns
Δ run length (μm)
H
WT or G2019S KI
mouse cortical neurons
MLi-2 (or DMSO)
Mito-SNAP
DMSO-treated mouse neurons
WT
G2019S
60 s
10 μm
(retrograde)
I
mitos / 30 μm axon
ns
MLi-2
WT
G2019S
J
mito length (μm)
ns
K
fraction of mitos
ns
antero.
retro.
bidir./stat.
DMSO
MLi-2
WT
G2019S

Figure S6. LRRK2-p.G2019S does not disrupt axonal mitochondrial transport in human iNeurons or mouse cortical neurons. Related to Figure 4. (A) Kymographs of axonal mitochondria labeled by Mito-mEmerald in WT and p.G2019S KI iNeurons. Example mitochondrial traces are highlighted. (B) Directionality of mitochondria in WT and p.G2019S KI iNeurons. Antero., anterograde; retro., retrograde; bidir., bi-directional; stat., stationary (mean ± SEM; n = 59-60 neurons from 4 independent experiments; ns, not significant, $p>0.6058$; **$p=0.0012$; two-way ANOVA with Sidak's multiple comparisons test). (C-D) Density per 30 μm axon (C) and average length (D) of total population of mitochondria in WT and p.G2019S KI iNeurons (mean ± SD; n = 833-1015 mitochondria from 59-60 neurons from 4 independent experiments; ns, not significant, $p>0.1846$; *$p=0.0105$; mixed effects model analysis). (E-G) Pause number (E), pause duration (F), and Δ run length (G) of mitochondria in WT and p.G2019S KI iNeurons (mean ± SD for panels E-F; n = 186-317 motile mitochondria from 59-60 neurons from 4 independent experiments; ns, not significant, $p>0.265$; mixed effects model analysis). (H) Kymographs of axonal mitochondria labeled by Mito-SNAP in WT and p.G2019S KI mouse cortical neurons treated with DMSO. (I-J) Density per 30 μm axon (I) and average length (J) of mitochondria in WT and p.G2019S KI mouse cortical neurons treated overnight with DMSO or 100 nM MLi-2 (mean ± SD; n = 97-131 mitochondria from 18-21 neurons from 3 independent experiments; ns, not significant, $p>0.1924$; mixed effects model analysis). Scatter plot points indicate the means of three independent experiments. (K) Directionality of mitochondria in WT and p.G2019S KI mouse cortical neurons treated with DMSO or MLi-2. Antero., anterograde; retro., retrograde; bidir./stat., bi-directional or stationary (mean ± SEM; n = 18-21 neurons from 3 independent experiments; ns, not significant, $p>0.4972$; two-way ANOVA with Sidak's multiple comparisons test). For panels C-F and I-J, scatter plot points indicate the means of three independent experiments. For panel G, a curve fit was generated using nonlinear regression (two phase decay).
